# Supplementary material for: MetaFunPrimer: an Environment-Specific, High-Throughput Primer Design Tool for Improved Quantification of Target Genes
Source: mSystems. 2021 Sep 21;6(5):e00201-21. doi: 10.1128/mSystems.00201-21 (PMC8547451; doi:10.1128/mSystems.00201-21)
Supplement: FIG S1 [file msystems.00201-21-sf001.docx]

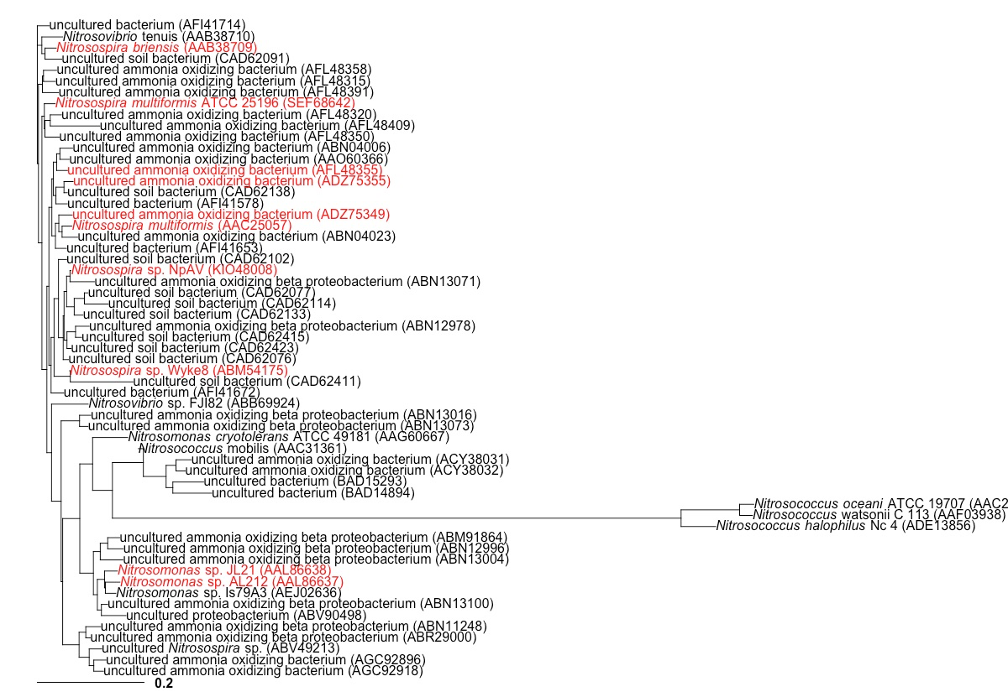


**Fig. S1**Phylogenetic tree of 60 *amo*A-AOB cluster representative genes used for primer design. The representatives of the 60 gene clusters found after clustering the input *amo*A-AOB sequences at 96% amino acid sequence similarity were aligned using MAFFT (1) and a tree was constructed using the NGPhylogeny.fr online service (2). Red labels indicate those clusters whose cumulative R-score totaled greater than 80% after ordering genes by R-score.

**REFERENCES**

1. Katoh K, Standley DM. 2013. MAFFT multiple sequence alignment software version 7: Improvements in performance and usability. Mol Biol Evol 30:772–780.

2. Dereeper A, Guignon V, Blanc G, Audic S, Buffet S, Chevenet F, Dufayard JF, Guindon S, Lefort V, Lescot M, Claverie JM, Gascuel O. 2008. Phylogeny.fr: robust phylogenetic analysis for the non-specialist. Nucleic Acids Res doi: 10.1093/nar/gkn180.
