# Supplementary material for: MetaFunPrimer: an Environment-Specific, High-Throughput Primer Design Tool for Improved Quantification of Target Genes
Source: mSystems. 2021 Sep 21;6(5):e00201-21. doi: 10.1128/mSystems.00201-21 (PMC8547451; doi:10.1128/mSystems.00201-21)
Supplement: TABLE S1 [file msystems.00201-21-st001.docx]

**TABLE S1**Publicly available soil metagenomes used in this study. Metagenomic data were downloaded from the Metagenomics RAST Server (http://metagenomics.nmpdr.org) and the Genome Portal of the Department of Energy Joint Genome Institute (http://genome.jgi.doe.gov).

| **Sample Name** | **MG-RAST ID** | **JGI Project ID** |
| --- | --- | --- |
| mgm4477804.3 | 4477804.3 |  |
| mgm4477805.3 | 4477805.3 |  |
| mgm4477872.3 | 4477872.3 |  |
| mgm4477873.3 | 4477873.3 |  |
| mgm4477877.3 | 4477877.3 |  |
| mgm4477899.3 | 4477899.3 |  |
| mgm4482599.3 | 4482599.3 |  |
| mgm4482600.3 | 4482600.3 |  |
| mgm4483819.3 | 4483819.3 |  |
| mgm4483820.3 | 4483820.3 |  |
| mgm4492602.3 | 4492602.3 |  |
| mgm4492603.3 | 4492603.3 |  |
| mgm4492604.3 | 4492604.3 |  |
| mgm4492612.3 | 4492612.3 |  |
| mgm4492614.3 | 4492614.3 |  |
| mgm4492618.3 | 4492618.3 |  |
| mgm4492621.3 | 4492621.3 |  |
| mgm4492622.3 | 4492622.3 |  |
| mgm4492749.3 | 4492749.3 |  |
| mgm4492750.3 | 4492750.3 |  |
| mgm4492757.3 | 4492757.3 |  |
| mgm4492760.3 | 4492760.3 |  |
| mgm4492761.3 | 4492761.3 |  |
| mgm4492762.3 | 4492762.3 |  |
| mgm4496754.3 | 4496754.3 |  |
| mgm4502539.3 | 4502539.3 |  |
| mgm4502540.3 | 4502540.3 |  |
| mgm4502541.3 | 4502541.3 |  |
| mgm4502542.3 | 4502542.3 |  |
| mgm4502543.3 | 4502543.3 |  |
| mgm4502923.3 | 4502923.3 |  |
| mgm4502924.3 | 4502924.3 |  |
| mgm4502925.3 | 4502925.3 |  |
| mgm4502926.3 | 4502926.3 |  |
| mgm4502927.3 | 4502927.3 |  |
| mgm4502928.3 | 4502928.3 |  |
| mgm4502929.3 | 4502929.3 |  |
| mgm4502930.3 | 4502930.3 |  |
| mgm4502931.3 | 4502931.3 |  |
| mgm4502932.3 | 4502932.3 |  |
| mgm4502933.3 | 4502933.3 |  |
| mgm4502934.3 | 4502934.3 |  |
| mgm4502935.3 | 4502935.3 |  |
| mgm4508937.3 | 4508937.3 |  |
| mgm4508938.3 | 4508938.3 |  |
| mgm4508939.3 | 4508939.3 |  |
| mgm4508940.3 | 4508940.3 |  |
| mgm4508941.3 | 4508941.3 |  |
| mgm4508942.3 | 4508942.3 |  |
| mgm4509396.3 | 4509396.3 |  |
| mgm4509397.3 | 4509397.3 |  |
| mgm4509398.3 | 4509398.3 |  |
| mgm4509399.3 | 4509399.3 |  |
| mgm4509400.3 | 4509400.3 |  |
| mgm4509401.3 | 4509401.3 |  |
| mgm4509402.3 | 4509402.3 |  |
| mgm4509403.3 | 4509403.3 |  |
| mgm4509404.3 | 4509404.3 |  |
| mgm4509405.3 | 4509405.3 |  |
| mgm4509406.3 | 4509406.3 |  |
| mgm4509407.3 | 4509407.3 |  |
| mgm4511045.3 | 4511045.3 |  |
| mgm4511046.3 | 4511046.3 |  |
| mgm4511047.3 | 4511047.3 |  |
| mgm4511048.3 | 4511048.3 |  |
| mgm4511049.3 | 4511049.3 |  |
| mgm4511050.3 | 4511050.3 |  |
| mgm4511060.3 | 4511060.3 |  |
| mgm4511061.3 | 4511061.3 |  |
| mgm4511062.3 | 4511062.3 |  |
| mgm4511063.3 | 4511063.3 |  |
| mgm4511064.3 | 4511064.3 |  |
| mgm4511065.3 | 4511065.3 |  |
| mgm4511111.3 | 4511111.3 |  |
| mgm4511112.3 | 4511112.3 |  |
| mgm4511113.3 | 4511113.3 |  |
| mgm4511114.3 | 4511114.3 |  |
| mgm4511115.3 | 4511115.3 |  |
| mgm4511116.3 | 4511116.3 |  |
| mgm4511134.3 | 4511134.3 |  |
| mgm4511135.3 | 4511135.3 |  |
| mgm4511136.3 | 4511136.3 |  |
| mgm4511137.3 | 4511137.3 |  |
| mgm4511138.3 | 4511138.3 |  |
| mgm4511139.3 | 4511139.3 |  |
| mgm4511140.3 | 4511140.3 |  |
| mgm4511141.3 | 4511141.3 |  |
| mgm4511142.3 | 4511142.3 |  |
| mgm4511143.3 | 4511143.3 |  |
| mgm4511144.3 | 4511144.3 |  |
| mgm4511145.3 | 4511145.3 |  |
| mgm4511146.3 | 4511146.3 |  |
| mgm4511147.3 | 4511147.3 |  |
| mgm4511148.3 | 4511148.3 |  |
| mgm4511149.3 | 4511149.3 |  |
| mgm4511150.3 | 4511150.3 |  |
| mgm4511151.3 | 4511151.3 |  |
| mgm4511152.3 | 4511152.3 |  |
| mgm4511153.3 | 4511153.3 |  |
| mgm4511173.3 | 4511173.3 |  |
| mgm4511174.3 | 4511174.3 |  |
| mgm4511175.3 | 4511175.3 |  |
| mgm4511176.3 | 4511176.3 |  |
| mgm4511178.3 | 4511178.3 |  |
| mgm4511179.3 | 4511179.3 |  |
| mgm4511180.3 | 4511180.3 |  |
| mgm4511181.3 | 4511181.3 |  |
| mgm4511182.3 | 4511182.3 |  |
| mgm4511183.3 | 4511183.3 |  |
| mgm4511184.3 | 4511184.3 |  |
| mgm4511185.3 | 4511185.3 |  |
| mgm4511186.3 | 4511186.3 |  |
| mgm4511187.3 | 4511187.3 |  |
| mgm4511188.3 | 4511188.3 |  |
| mgm4511189.3 | 4511189.3 |  |
| mgm4511190.3 | 4511190.3 |  |
| mgm4511191.3 | 4511191.3 |  |
| mgm4511192.3 | 4511192.3 |  |
| mgm4511193.3 | 4511193.3 |  |
| mgm4514321.3 | 4514321.3 |  |
| mgm4514322.3 | 4514322.3 |  |
| mgm4514323.3 | 4514323.3 |  |
| mgm4514324.3 | 4514324.3 |  |
| mgm4514325.3 | 4514325.3 |  |
| mgm4514326.3 | 4514326.3 |  |
| mgm4514327.3 | 4514327.3 |  |
| mgm4514328.3 | 4514328.3 |  |
| mgm4514329.3 | 4514329.3 |  |
| mgm4514330.3 | 4514330.3 |  |
| mgm4514331.3 | 4514331.3 |  |
| mgm4520300.3 | 4520300.3 |  |
| mgm4520301.3 | 4520301.3 |  |
| mgm4520302.3 | 4520302.3 |  |
| mgm4520303.3 | 4520303.3 |  |
| mgm4520304.3 | 4520304.3 |  |
| mgm4520305.3 | 4520305.3 |  |
| mgm4520306.3 | 4520306.3 |  |
| mgm4520307.3 | 4520307.3 |  |
| mgm4520308.3 | 4520308.3 |  |
| mgm4520309.3 | 4520309.3 |  |
| mgm4520310.3 | 4520310.3 |  |
| mgm4520311.3 | 4520311.3 |  |
| mgm4520312.3 | 4520312.3 |  |
| mgm4520313.3 | 4520313.3 |  |
| mgm4520314.3 | 4520314.3 |  |
| mgm4520315.3 | 4520315.3 |  |
| mgm4520316.3 | 4520316.3 |  |
| mgm4520317.3 | 4520317.3 |  |
| mgm4520318.3 | 4520318.3 |  |
| mgm4520319.3 | 4520319.3 |  |
| mgm4520320.3 | 4520320.3 |  |
| mgm4520321.3 | 4520321.3 |  |
| mgm4520322.3 | 4520322.3 |  |
| mgm4520323.3 | 4520323.3 |  |
| mgm4520324.3 | 4520324.3 |  |
| mgm4520325.3 | 4520325.3 |  |
| mgm4520326.3 | 4520326.3 |  |
| mgm4520327.3 | 4520327.3 |  |
| mgm4520328.3 | 4520328.3 |  |
| mgm4520329.3 | 4520329.3 |  |
| mgm4520330.3 | 4520330.3 |  |
| mgm4520331.3 | 4520331.3 |  |
| mgm4520332.3 | 4520332.3 |  |
| mgm4520333.3 | 4520333.3 |  |
| mgm4520334.3 | 4520334.3 |  |
| mgm4520335.3 | 4520335.3 |  |
| mgm4520336.3 | 4520336.3 |  |
| mgm4520337.3 | 4520337.3 |  |
| mgm4520338.3 | 4520338.3 |  |
| mgm4520339.3 | 4520339.3 |  |
| mgm4520340.3 | 4520340.3 |  |
| mgm4520341.3 | 4520341.3 |  |
| mgm4520342.3 | 4520342.3 |  |
| mgm4520343.3 | 4520343.3 |  |
| mgm4520344.3 | 4520344.3 |  |
| mgm4520345.3 | 4520345.3 |  |
| mgm4520346.3 | 4520346.3 |  |
| mgm4520347.3 | 4520347.3 |  |
| mgm4520348.3 | 4520348.3 |  |
| mgm4520349.3 | 4520349.3 |  |
| mgm4520350.3 | 4520350.3 |  |
| mgm4520351.3 | 4520351.3 |  |
| mgm4520352.3 | 4520352.3 |  |
| mgm4520353.3 | 4520353.3 |  |
| mgm4520354.3 | 4520354.3 |  |
| mgm4520355.3 | 4520355.3 |  |
| mgm4520356.3 | 4520356.3 |  |
| mgm4520357.3 | 4520357.3 |  |
| mgm4520358.3 | 4520358.3 |  |
| mgm4520359.3 | 4520359.3 |  |
| mgm4520360.3 | 4520360.3 |  |
| mgm4520361.3 | 4520361.3 |  |
| mgm4520362.3 | 4520362.3 |  |
| mgm4520363.3 | 4520363.3 |  |
| mgm4520364.3 | 4520364.3 |  |
| mgm4520365.3 | 4520365.3 |  |
| mgm4520366.3 | 4520366.3 |  |
| mgm4520367.3 | 4520367.3 |  |
| mgm4520368.3 | 4520368.3 |  |
| mgm4520369.3 | 4520369.3 |  |
| mgm4520370.3 | 4520370.3 |  |
| mgm4520371.3 | 4520371.3 |  |
| mgm4520372.3 | 4520372.3 |  |
| mgm4520373.3 | 4520373.3 |  |
| mgm4520374.3 | 4520374.3 |  |
| mgm4520375.3 | 4520375.3 |  |
| mgm4520376.3 | 4520376.3 |  |
| mgm4520377.3 | 4520377.3 |  |
| mgm4520378.3 | 4520378.3 |  |
| mgm4520379.3 | 4520379.3 |  |
| mgm4520380.3 | 4520380.3 |  |
| mgm4520381.3 | 4520381.3 |  |
| mgm4520382.3 | 4520382.3 |  |
| mgm4520383.3 | 4520383.3 |  |
| mgm4520384.3 | 4520384.3 |  |
| mgm4520385.3 | 4520385.3 |  |
| mgm4520386.3 | 4520386.3 |  |
| mgm4520387.3 | 4520387.3 |  |
| mgm4520388.3 | 4520388.3 |  |
| mgm4520389.3 | 4520389.3 |  |
| mgm4520390.3 | 4520390.3 |  |
| mgm4520391.3 | 4520391.3 |  |
| mgm4520392.3 | 4520392.3 |  |
| mgm4520393.3 | 4520393.3 |  |
| mgm4521317.3 | 4521317.3 |  |
| mgm4523238.3 | 4523238.3 |  |
| mgm4523239.3 | 4523239.3 |  |
| mgm4523240.3 | 4523240.3 |  |
| mgm4529642.3 | 4529642.3 |  |
| mgm4529643.3 | 4529643.3 |  |
| mgm4529644.3 | 4529644.3 |  |
| mgm4529645.3 | 4529645.3 |  |
| mgm4529646.3 | 4529646.3 |  |
| mgm4529647.3 | 4529647.3 |  |
| mgm4529648.3 | 4529648.3 |  |
| mgm4529649.3 | 4529649.3 |  |
| mgm4529650.3 | 4529650.3 |  |
| mgm4529651.3 | 4529651.3 |  |
| mgm4529652.3 | 4529652.3 |  |
| mgm4529653.3 | 4529653.3 |  |
| mgm4529654.3 | 4529654.3 |  |
| mgm4529655.3 | 4529655.3 |  |
| mgm4529656.3 | 4529656.3 |  |
| mgm4529657.3 | 4529657.3 |  |
| mgm4529733.3 | 4529733.3 |  |
| mgm4529734.3 | 4529734.3 |  |
| mgm4529735.3 | 4529735.3 |  |
| mgm4529736.3 | 4529736.3 |  |
| mgm4529737.3 | 4529737.3 |  |
| mgm4529738.3 | 4529738.3 |  |
| mgm4529739.3 | 4529739.3 |  |
| mgm4529740.3 | 4529740.3 |  |
| mgm4529741.3 | 4529741.3 |  |
| mgm4529742.3 | 4529742.3 |  |
| mgm4529743.3 | 4529743.3 |  |
| mgm4529744.3 | 4529744.3 |  |
| mgm4529836.3 | 4529836.3 |  |
| mgm4530504.3 | 4530504.3 |  |
| mgm4535613.3 | 4535613.3 |  |
| mgm4535614.3 | 4535614.3 |  |
| mgm4535615.3 | 4535615.3 |  |
| mgm4535616.3 | 4535616.3 |  |
| mgm4535617.3 | 4535617.3 |  |
| mgm4535618.3 | 4535618.3 |  |
| mgm4535619.3 | 4535619.3 |  |
| mgm4535620.3 | 4535620.3 |  |
| mgm4535621.3 | 4535621.3 |  |
| mgm4535622.3 | 4535622.3 |  |
| mgm4535623.3 | 4535623.3 |  |
| mgm4535624.3 | 4535624.3 |  |
| mgm4535625.3 | 4535625.3 |  |
| mgm4537557.3 | 4537557.3 |  |
| mgm4537558.3 | 4537558.3 |  |
| mgm4537559.3 | 4537559.3 |  |
| mgm4537560.3 | 4537560.3 |  |
| mgm4537561.3 | 4537561.3 |  |
| mgm4537562.3 | 4537562.3 |  |
| mgm4537564.3 | 4537564.3 |  |
| mgm4537565.3 | 4537565.3 |  |
| mgm4537566.3 | 4537566.3 |  |
| mgm4537567.3 | 4537567.3 |  |
| mgm4538627.3 | 4538627.3 |  |
| mgm4538778.3 | 4538778.3 |  |
| mgm4538779.3 | 4538779.3 |  |
| mgm4538997.3 | 4538997.3 |  |
| mgm4539063.3 | 4539063.3 |  |
| mgm4539064.3 | 4539064.3 |  |
| mgm4539517.3 | 4539517.3 |  |
| mgm4539518.3 | 4539518.3 |  |
| mgm4539519.3 | 4539519.3 |  |
| mgm4539520.3 | 4539520.3 |  |
| mgm4539521.3 | 4539521.3 |  |
| mgm4539522.3 | 4539522.3 |  |
| mgm4539523.3 | 4539523.3 |  |
| mgm4539524.3 | 4539524.3 |  |
| mgm4539525.3 | 4539525.3 |  |
| mgm4539526.3 | 4539526.3 |  |
| mgm4539527.3 | 4539527.3 |  |
| mgm4539571.3 | 4539571.3 |  |
| mgm4539572.3 | 4539572.3 |  |
| mgm4539573.3 | 4539573.3 |  |
| mgm4539574.3 | 4539574.3 |  |
| mgm4539575.3 | 4539575.3 |  |
| mgm4539576.3 | 4539576.3 |  |
| mgm4539577.3 | 4539577.3 |  |
| mgm4539578.3 | 4539578.3 |  |
| mgm4539579.3 | 4539579.3 |  |
| mgm4539580.3 | 4539580.3 |  |
| mgm4539581.3 | 4539581.3 |  |
| mgm4539582.3 | 4539582.3 |  |
| mgm4539583.3 | 4539583.3 |  |
| mgm4539584.3 | 4539584.3 |  |
| mgm4539585.3 | 4539585.3 |  |
| mgm4539586.3 | 4539586.3 |  |
| mgm4539587.3 | 4539587.3 |  |
| mgm4539588.3 | 4539588.3 |  |
| mgm4539589.3 | 4539589.3 |  |
| mgm4539590.3 | 4539590.3 |  |
| mgm4539591.3 | 4539591.3 |  |
| mgm4539592.3 | 4539592.3 |  |
| mgm4539593.3 | 4539593.3 |  |
| mgm4539594.3 | 4539594.3 |  |
| mgm4541641.3 | 4541641.3 |  |
| mgm4541642.3 | 4541642.3 |  |
| mgm4541644.3 | 4541644.3 |  |
| mgm4541645.3 | 4541645.3 |  |
| mgm4541646.3 | 4541646.3 |  |
| mgm4541647.3 | 4541647.3 |  |
| mgm4541648.3 | 4541648.3 |  |
| mgm4541649.3 | 4541649.3 |  |
| mgm4541650.3 | 4541650.3 |  |
| mgm4541651.3 | 4541651.3 |  |
| mgm4547279.3 | 4547279.3 |  |
| mgm4547280.3 | 4547280.3 |  |
| mgm4547281.3 | 4547281.3 |  |
| mgm4547282.3 | 4547282.3 |  |
| mgm4547283.3 | 4547283.3 |  |
| mgm4547284.3 | 4547284.3 |  |
| mgm4547285.3 | 4547285.3 |  |
| mgm4552597.3 | 4552597.3 |  |
| mgm4552598.3 | 4552598.3 |  |
| mgm4552599.3 | 4552599.3 |  |
| mgm4552600.3 | 4552600.3 |  |
| mgm4552601.3 | 4552601.3 |  |
| mgm4552602.3 | 4552602.3 |  |
| mgm4552603.3 | 4552603.3 |  |
| mgm4552604.3 | 4552604.3 |  |
| mgm4552605.3 | 4552605.3 |  |
| mgm4552606.3 | 4552606.3 |  |
| mgm4552607.3 | 4552607.3 |  |
| mgm4552608.3 | 4552608.3 |  |
| mgm4552609.3 | 4552609.3 |  |
| mgm4552610.3 | 4552610.3 |  |
| mgm4552611.3 | 4552611.3 |  |
| mgm4552612.3 | 4552612.3 |  |
| mgm4552613.3 | 4552613.3 |  |
| mgm4552614.3 | 4552614.3 |  |
| mgm4552615.3 | 4552615.3 |  |
| mgm4552616.3 | 4552616.3 |  |
| mgm4552617.3 | 4552617.3 |  |
| mgm4552618.3 | 4552618.3 |  |
| mgm4552619.3 | 4552619.3 |  |
| mgm4552620.3 | 4552620.3 |  |
| mgm4552621.3 | 4552621.3 |  |
| mgm4552622.3 | 4552622.3 |  |
| mgm4552623.3 | 4552623.3 |  |
| mgm4552624.3 | 4552624.3 |  |
| mgm4552625.3 | 4552625.3 |  |
| mgm4552626.3 | 4552626.3 |  |
| mgm4552627.3 | 4552627.3 |  |
| mgm4552628.3 | 4552628.3 |  |
| mgm4552629.3 | 4552629.3 |  |
| mgm4552630.3 | 4552630.3 |  |
| mgm4552631.3 | 4552631.3 |  |
| mgm4552632.3 | 4552632.3 |  |
| mgm4552633.3 | 4552633.3 |  |
| mgm4552634.3 | 4552634.3 |  |
| mgm4552635.3 | 4552635.3 |  |
| mgm4552636.3 | 4552636.3 |  |
| mgm4554763.3 | 4554763.3 |  |
| mgm4554764.3 | 4554764.3 |  |
| mgm4554765.3 | 4554765.3 |  |
| mgm4554766.3 | 4554766.3 |  |
| mgm4554767.3 | 4554767.3 |  |
| mgm4554768.3 | 4554768.3 |  |
| mgm4554769.3 | 4554769.3 |  |
| mgm4554770.3 | 4554770.3 |  |
| mgm4554771.3 | 4554771.3 |  |
| mgm4573678.3 | 4573678.3 |  |
| mgm4573679.3 | 4573679.3 |  |
| mgm4573680.3 | 4573680.3 |  |
| mgm4573681.3 | 4573681.3 |  |
| mgm4573682.3 | 4573682.3 |  |
| mgm4573683.3 | 4573683.3 |  |
| mgm4583642.3 | 4583642.3 |  |
| mgm4583643.3 | 4583643.3 |  |
| mgm4583644.3 | 4583644.3 |  |
| mgm4583645.3 | 4583645.3 |  |
| mgm4583646.3 | 4583646.3 |  |
| mgm4583647.3 | 4583647.3 |  |
| mgm4583648.3 | 4583648.3 |  |
| mgm4583649.3 | 4583649.3 |  |
| mgm4584765.3 | 4584765.3 |  |
| mgm4584766.3 | 4584766.3 |  |
| mgm4584767.3 | 4584767.3 |  |
| mgm4584768.3 | 4584768.3 |  |
| mgm4584769.3 | 4584769.3 |  |
| mgm4584770.3 | 4584770.3 |  |
| mgm4584771.3 | 4584771.3 |  |
| mgm4584873.3 | 4584873.3 |  |
| mgm4600900.3 | 4600900.3 |  |
| mgm4600901.3 | 4600901.3 |  |
| mgm4600902.3 | 4600902.3 |  |
| mgm4600903.3 | 4600903.3 |  |
| mgm4600904.3 | 4600904.3 |  |
| mgm4600905.3 | 4600905.3 |  |
| mgm4600906.3 | 4600906.3 |  |
| mgm4600907.3 | 4600907.3 |  |
| mgm4600908.3 | 4600908.3 |  |
| mgm4600909.3 | 4600909.3 |  |
| mgm4600910.3 | 4600910.3 |  |
| mgm4600911.3 | 4600911.3 |  |
| mgm4612650.3 | 4612650.3 |  |
| mgm4612651.3 | 4612651.3 |  |
| mgm4612652.3 | 4612652.3 |  |
| mgm4612653.3 | 4612653.3 |  |
| mgm4612654.3 | 4612654.3 |  |
| mgm4612655.3 | 4612655.3 |  |
| mgm4612987.3 | 4612987.3 |  |
| mgm4612988.3 | 4612988.3 |  |
| mgm4614815.3 | 4614815.3 |  |
| mgm4614816.3 | 4614816.3 |  |
| mgm4614817.3 | 4614817.3 |  |
| mgm4614818.3 | 4614818.3 |  |
| mgm4614819.3 | 4614819.3 |  |
| mgm4614820.3 | 4614820.3 |  |
| mgm4614821.3 | 4614821.3 |  |
| mgm4614822.3 | 4614822.3 |  |
| mgm4614823.3 | 4614823.3 |  |
| mgm4614824.3 | 4614824.3 |  |
| mgm4614825.3 | 4614825.3 |  |
| mgm4614826.3 | 4614826.3 |  |
| mgm4614827.3 | 4614827.3 |  |
| mgm4614828.3 | 4614828.3 |  |
| mgm4614829.3 | 4614829.3 |  |
| mgm4614830.3 | 4614830.3 |  |
| mgm4614831.3 | 4614831.3 |  |
| mgm4614832.3 | 4614832.3 |  |
| mgm4614833.3 | 4614833.3 |  |
| mgm4614834.3 | 4614834.3 |  |
| mgm4614835.3 | 4614835.3 |  |
| mgm4614836.3 | 4614836.3 |  |
| mgm4614837.3 | 4614837.3 |  |
| mgm4614838.3 | 4614838.3 |  |
| mgm4620526.3 | 4620526.3 |  |
| mgm4620527.3 | 4620527.3 |  |
| mgm4620528.3 | 4620528.3 |  |
| mgm4620529.3 | 4620529.3 |  |
| mgm4620530.3 | 4620530.3 |  |
| mgm4620531.3 | 4620531.3 |  |
| mgm4620532.3 | 4620532.3 |  |
| mgm4620533.3 | 4620533.3 |  |
| mgm4620534.3 | 4620534.3 |  |
| mgm4620535.3 | 4620535.3 |  |
| mgm4620536.3 | 4620536.3 |  |
| mgm4620537.3 | 4620537.3 |  |
| mgm4621360.3 | 4621360.3 |  |
| mgm4621361.3 | 4621361.3 |  |
| mgm4621362.3 | 4621362.3 |  |
| mgm4621363.3 | 4621363.3 |  |
| mgm4621364.3 | 4621364.3 |  |
| mgm4621365.3 | 4621365.3 |  |
| mgm4622488.3 | 4622488.3 |  |
| mgm4622489.3 | 4622489.3 |  |
| mgm4622490.3 | 4622490.3 |  |
| mgm4623639.3 | 4623639.3 |  |
| mgm4623640.3 | 4623640.3 |  |
| mgm4623641.3 | 4623641.3 |  |
| mgm4623642.3 | 4623642.3 |  |
| mgm4623643.3 | 4623643.3 |  |
| mgm4623644.3 | 4623644.3 |  |
| mgm4623645.3 | 4623645.3 |  |
| mgm4623646.3 | 4623646.3 |  |
| mgm4623647.3 | 4623647.3 |  |
| mgm4623648.3 | 4623648.3 |  |
| mgm4623649.3 | 4623649.3 |  |
| mgm4623650.3 | 4623650.3 |  |
| mgm4623651.3 | 4623651.3 |  |
| mgm4623652.3 | 4623652.3 |  |
| mgm4623653.3 | 4623653.3 |  |
| mgm4623654.3 | 4623654.3 |  |
| mgm4623655.3 | 4623655.3 |  |
| mgm4623656.3 | 4623656.3 |  |
| mgm4623657.3 | 4623657.3 |  |
| mgm4623658.3 | 4623658.3 |  |
| mgm4623659.3 | 4623659.3 |  |
| mgm4623660.3 | 4623660.3 |  |
| mgm4623661.3 | 4623661.3 |  |
| mgm4623662.3 | 4623662.3 |  |
| mgm4623663.3 | 4623663.3 |  |
| mgm4623664.3 | 4623664.3 |  |
| mgm4623665.3 | 4623665.3 |  |
| mgm4623666.3 | 4623666.3 |  |
| mgm4623667.3 | 4623667.3 |  |
| mgm4623668.3 | 4623668.3 |  |
| mgm4623669.3 | 4623669.3 |  |
| mgm4623670.3 | 4623670.3 |  |
| mgm4623671.3 | 4623671.3 |  |
| mgm4623672.3 | 4623672.3 |  |
| mgm4623673.3 | 4623673.3 |  |
| mgm4623674.3 | 4623674.3 |  |
| mgm4623675.3 | 4623675.3 |  |
| mgm4623676.3 | 4623676.3 |  |
| mgm4623677.3 | 4623677.3 |  |
| mgm4623678.3 | 4623678.3 |  |
| mgm4623679.3 | 4623679.3 |  |
| mgm4623680.3 | 4623680.3 |  |
| mgm4623681.3 | 4623681.3 |  |
| mgm4623682.3 | 4623682.3 |  |
| mgm4623683.3 | 4623683.3 |  |
| mgm4623684.3 | 4623684.3 |  |
| mgm4623685.3 | 4623685.3 |  |
| mgm4623686.3 | 4623686.3 |  |
| mgm4623687.3 | 4623687.3 |  |
| mgm4623688.3 | 4623688.3 |  |
| mgm4623689.3 | 4623689.3 |  |
| mgm4623690.3 | 4623690.3 |  |
| mgm4623691.3 | 4623691.3 |  |
| mgm4623692.3 | 4623692.3 |  |
| mgm4623693.3 | 4623693.3 |  |
| mgm4623694.3 | 4623694.3 |  |
| mgm4623695.3 | 4623695.3 |  |
| mgm4623696.3 | 4623696.3 |  |
| mgm4623697.3 | 4623697.3 |  |
| mgm4623698.3 | 4623698.3 |  |
| mgm4623699.3 | 4623699.3 |  |
| mgm4623700.3 | 4623700.3 |  |
| mgm4623701.3 | 4623701.3 |  |
| mgm4623702.3 | 4623702.3 |  |
| mgm4626743.3 | 4626743.3 |  |
| mgm4626744.3 | 4626744.3 |  |
| mgm4626745.3 | 4626745.3 |  |
| mgm4626746.3 | 4626746.3 |  |
| mgm4626747.3 | 4626747.3 |  |
| mgm4626748.3 | 4626748.3 |  |
| mgm4626753.3 | 4626753.3 |  |
| mgm4626754.3 | 4626754.3 |  |
| mgm4626755.3 | 4626755.3 |  |
| mgm4626756.3 | 4626756.3 |  |
| mgm4633076.3 | 4633076.3 |  |
| mgm4633077.3 | 4633077.3 |  |
| mgm4633078.3 | 4633078.3 |  |
| mgm4633079.3 | 4633079.3 |  |
| mgm4633080.3 | 4633080.3 |  |
| mgm4633081.3 | 4633081.3 |  |
| mgm4633082.3 | 4633082.3 |  |
| mgm4633083.3 | 4633083.3 |  |
| mgm4633084.3 | 4633084.3 |  |
| mgm4633085.3 | 4633085.3 |  |
| mgm4633086.3 | 4633086.3 |  |
| mgm4633087.3 | 4633087.3 |  |
| mgm4633088.3 | 4633088.3 |  |
| mgm4633089.3 | 4633089.3 |  |
| mgm4633090.3 | 4633090.3 |  |
| mgm4633091.3 | 4633091.3 |  |
| mgm4633092.3 | 4633092.3 |  |
| mgm4633093.3 | 4633093.3 |  |
| mgm4633094.3 | 4633094.3 |  |
| mgm4633095.3 | 4633095.3 |  |
| mgm4633096.3 | 4633096.3 |  |
| mgm4633097.3 | 4633097.3 |  |
| mgm4633098.3 | 4633098.3 |  |
| mgm4633099.3 | 4633099.3 |  |
| mgm4633100.3 | 4633100.3 |  |
| mgm4633101.3 | 4633101.3 |  |
| mgm4633102.3 | 4633102.3 |  |
| mgm4633103.3 | 4633103.3 |  |
| mgm4633104.3 | 4633104.3 |  |
| mgm4633105.3 | 4633105.3 |  |
| mgm4633106.3 | 4633106.3 |  |
| mgm4633107.3 | 4633107.3 |  |
| mgm4633108.3 | 4633108.3 |  |
| mgm4633109.3 | 4633109.3 |  |
| mgm4633110.3 | 4633110.3 |  |
| mgm4633111.3 | 4633111.3 |  |
| mgm4633112.3 | 4633112.3 |  |
| mgm4633113.3 | 4633113.3 |  |
| mgm4633114.3 | 4633114.3 |  |
| mgm4633115.3 | 4633115.3 |  |
| mgm4633116.3 | 4633116.3 |  |
| mgm4633117.3 | 4633117.3 |  |
| mgm4633118.3 | 4633118.3 |  |
| mgm4633119.3 | 4633119.3 |  |
| mgm4633120.3 | 4633120.3 |  |
| mgm4633121.3 | 4633121.3 |  |
| mgm4633122.3 | 4633122.3 |  |
| mgm4633188.3 | 4633188.3 |  |
| mgm4633189.3 | 4633189.3 |  |
| mgm4635362.3 | 4635362.3 |  |
| mgm4635608.3 | 4635608.3 |  |
| mgm4635609.3 | 4635609.3 |  |
| mgm4635904.3 | 4635904.3 |  |
| mgm4635905.3 | 4635905.3 |  |
| mgm4636837.3 | 4636837.3 |  |
| mgm4637809.3 | 4637809.3 |  |
| mgm4637810.3 | 4637810.3 |  |
| mgm4637811.3 | 4637811.3 |  |
| mgm4637812.3 | 4637812.3 |  |
| mgm4637813.3 | 4637813.3 |  |
| mgm4637814.3 | 4637814.3 |  |
| mgm4637815.3 | 4637815.3 |  |
| mgm4637816.3 | 4637816.3 |  |
| mgm4637817.3 | 4637817.3 |  |
| mgm4637818.3 | 4637818.3 |  |
| mgm4637819.3 | 4637819.3 |  |
| mgm4637820.3 | 4637820.3 |  |
| mgm4637821.3 | 4637821.3 |  |
| mgm4637822.3 | 4637822.3 |  |
| mgm4637823.3 | 4637823.3 |  |
| mgm4637824.3 | 4637824.3 |  |
| mgm4637825.3 | 4637825.3 |  |
| mgm4637826.3 | 4637826.3 |  |
| mgm4637827.3 | 4637827.3 |  |
| mgm4637828.3 | 4637828.3 |  |
| mgm4637829.3 | 4637829.3 |  |
| mgm4637830.3 | 4637830.3 |  |
| mgm4637831.3 | 4637831.3 |  |
| mgm4637832.3 | 4637832.3 |  |
| mgm4637833.3 | 4637833.3 |  |
| mgm4637834.3 | 4637834.3 |  |
| mgm4637835.3 | 4637835.3 |  |
| mgm4637836.3 | 4637836.3 |  |
| mgm4637837.3 | 4637837.3 |  |
| mgm4637838.3 | 4637838.3 |  |
| mgm4637839.3 | 4637839.3 |  |
| mgm4637840.3 | 4637840.3 |  |
| mgm4637841.3 | 4637841.3 |  |
| mgm4637842.3 | 4637842.3 |  |
| mgm4637843.3 | 4637843.3 |  |
| mgm4637844.3 | 4637844.3 |  |
| mgm4637845.3 | 4637845.3 |  |
| mgm4637846.3 | 4637846.3 |  |
| mgm4637847.3 | 4637847.3 |  |
| mgm4637848.3 | 4637848.3 |  |
| mgm4637849.3 | 4637849.3 |  |
| mgm4637850.3 | 4637850.3 |  |
| mgm4637851.3 | 4637851.3 |  |
| mgm4637852.3 | 4637852.3 |  |
| mgm4637853.3 | 4637853.3 |  |
| mgm4637854.3 | 4637854.3 |  |
| mgm4637855.3 | 4637855.3 |  |
| mgm4637856.3 | 4637856.3 |  |
| mgm4637857.3 | 4637857.3 |  |
| mgm4637858.3 | 4637858.3 |  |
| mgm4637859.3 | 4637859.3 |  |
| mgm4637860.3 | 4637860.3 |  |
| mgm4637861.3 | 4637861.3 |  |
| mgm4637862.3 | 4637862.3 |  |
| mgm4637863.3 | 4637863.3 |  |
| mgm4637864.3 | 4637864.3 |  |
| mgm4637865.3 | 4637865.3 |  |
| mgm4637866.3 | 4637866.3 |  |
| mgm4637867.3 | 4637867.3 |  |
| mgm4637868.3 | 4637868.3 |  |
| mgm4653780.3 | 4653780.3 |  |
| mgm4653781.3 | 4653781.3 |  |
| mgm4653782.3 | 4653782.3 |  |
| mgm4653783.3 | 4653783.3 |  |
| mgm4653784.3 | 4653784.3 |  |
| mgm4653785.3 | 4653785.3 |  |
| mgm4653786.3 | 4653786.3 |  |
| mgm4653787.3 | 4653787.3 |  |
| mgm4653788.3 | 4653788.3 |  |
| mgm4653789.3 | 4653789.3 |  |
| mgm4653790.3 | 4653790.3 |  |
| mgm4653791.3 | 4653791.3 |  |
| mgm4654021.3 | 4654021.3 |  |
| mgm4654022.3 | 4654022.3 |  |
| mgm4654023.3 | 4654023.3 |  |
| mgm4654024.3 | 4654024.3 |  |
| mgm4654025.3 | 4654025.3 |  |
| mgm4654026.3 | 4654026.3 |  |
| mgm4654027.3 | 4654027.3 |  |
| mgm4654028.3 | 4654028.3 |  |
| mgm4654029.3 | 4654029.3 |  |
| mgm4654030.3 | 4654030.3 |  |
| mgm4654031.3 | 4654031.3 |  |
| mgm4654032.3 | 4654032.3 |  |
| mgm4664850.3 | 4664850.3 |  |
| mgm4664851.3 | 4664851.3 |  |
| mgm4664852.3 | 4664852.3 |  |
| mgm4664853.3 | 4664853.3 |  |
| mgm4664854.3 | 4664854.3 |  |
| mgm4664855.3 | 4664855.3 |  |
| mgm4664856.3 | 4664856.3 |  |
| mgm4664857.3 | 4664857.3 |  |
| mgm4664858.3 | 4664858.3 |  |
| mgm4664859.3 | 4664859.3 |  |
| mgm4664860.3 | 4664860.3 |  |
| mgm4664861.3 | 4664861.3 |  |
| mgm4664862.3 | 4664862.3 |  |
| mgm4664863.3 | 4664863.3 |  |
| mgm4664864.3 | 4664864.3 |  |
| mgm4664865.3 | 4664865.3 |  |
| mgm4664866.3 | 4664866.3 |  |
| mgm4664867.3 | 4664867.3 |  |
| mgm4664868.3 | 4664868.3 |  |
| mgm4664869.3 | 4664869.3 |  |
| mgm4664870.3 | 4664870.3 |  |
| mgm4664871.3 | 4664871.3 |  |
| mgm4664872.3 | 4664872.3 |  |
| mgm4664873.3 | 4664873.3 |  |
| mgm4664874.3 | 4664874.3 |  |
| mgm4664875.3 | 4664875.3 |  |
| mgm4664876.3 | 4664876.3 |  |
| mgm4664877.3 | 4664877.3 |  |
| mgm4664878.3 | 4664878.3 |  |
| mgm4664879.3 | 4664879.3 |  |
| mgm4664880.3 | 4664880.3 |  |
| mgm4664881.3 | 4664881.3 |  |
| mgm4664882.3 | 4664882.3 |  |
| mgm4664883.3 | 4664883.3 |  |
| mgm4664884.3 | 4664884.3 |  |
| mgm4664885.3 | 4664885.3 |  |
| mgm4664886.3 | 4664886.3 |  |
| mgm4664887.3 | 4664887.3 |  |
| mgm4664888.3 | 4664888.3 |  |
| mgm4664889.3 | 4664889.3 |  |
| mgm4664890.3 | 4664890.3 |  |
| mgm4664891.3 | 4664891.3 |  |
| mgm4664892.3 | 4664892.3 |  |
| mgm4664893.3 | 4664893.3 |  |
| mgm4664894.3 | 4664894.3 |  |
| mgm4664895.3 | 4664895.3 |  |
| mgm4664896.3 | 4664896.3 |  |
| mgm4664897.3 | 4664897.3 |  |
| mgm4664898.3 | 4664898.3 |  |
| mgm4664899.3 | 4664899.3 |  |
| mgm4664900.3 | 4664900.3 |  |
| mgm4664901.3 | 4664901.3 |  |
| mgm4664902.3 | 4664902.3 |  |
| mgm4664903.3 | 4664903.3 |  |
| mgm4664904.3 | 4664904.3 |  |
| mgm4664905.3 | 4664905.3 |  |
| mgm4664906.3 | 4664906.3 |  |
| mgm4664907.3 | 4664907.3 |  |
| mgm4664908.3 | 4664908.3 |  |
| mgm4664909.3 | 4664909.3 |  |
| mgm4664910.3 | 4664910.3 |  |
| mgm4664911.3 | 4664911.3 |  |
| mgm4664912.3 | 4664912.3 |  |
| mgm4664913.3 | 4664913.3 |  |
| mgm4664914.3 | 4664914.3 |  |
| mgm4664915.3 | 4664915.3 |  |
| mgm4664916.3 | 4664916.3 |  |
| mgm4664917.3 | 4664917.3 |  |
| mgm4664918.3 | 4664918.3 |  |
| mgm4664919.3 | 4664919.3 |  |
| mgm4664920.3 | 4664920.3 |  |
| mgm4664921.3 | 4664921.3 |  |
| mgm4664922.3 | 4664922.3 |  |
| mgm4664923.3 | 4664923.3 |  |
| mgm4664924.3 | 4664924.3 |  |
| mgm4664925.3 | 4664925.3 |  |
| mgm4664926.3 | 4664926.3 |  |
| mgm4664927.3 | 4664927.3 |  |
| mgm4664928.3 | 4664928.3 |  |
| mgm4664929.3 | 4664929.3 |  |
| mgm4664930.3 | 4664930.3 |  |
| mgm4664931.3 | 4664931.3 |  |
| mgm4667212.3 | 4667212.3 |  |
| mgm4667213.3 | 4667213.3 |  |
| mgm4667214.3 | 4667214.3 |  |
| mgm4667215.3 | 4667215.3 |  |
| mgm4667216.3 | 4667216.3 |  |
| mgm4667217.3 | 4667217.3 |  |
| mgm4667218.3 | 4667218.3 |  |
| mgm4667219.3 | 4667219.3 |  |
| mgm4667221.3 | 4667221.3 |  |
| mgm4667222.3 | 4667222.3 |  |
| mgm4667223.3 | 4667223.3 |  |
| mgm4667224.3 | 4667224.3 |  |
| mgm4667226.3 | 4667226.3 |  |
| mgm4667227.3 | 4667227.3 |  |
| mgm4667228.3 | 4667228.3 |  |
| mgm4667229.3 | 4667229.3 |  |
| mgm4667230.3 | 4667230.3 |  |
| mgm4667231.3 | 4667231.3 |  |
| mgm4667232.3 | 4667232.3 |  |
| mgm4667233.3 | 4667233.3 |  |
| mgm4667234.3 | 4667234.3 |  |
| mgm4667235.3 | 4667235.3 |  |
| mgm4667236.3 | 4667236.3 |  |
| mgm4667239.3 | 4667239.3 |  |
| mgm4667240.3 | 4667240.3 |  |
| mgm4667241.3 | 4667241.3 |  |
| mgm4670116.3 | 4670116.3 |  |
| mgm4670117.3 | 4670117.3 |  |
| mgm4670118.3 | 4670118.3 |  |
| mgm4670119.3 | 4670119.3 |  |
| mgm4670120.3 | 4670120.3 |  |
| mgm4670121.3 | 4670121.3 |  |
| mgm4670122.3 | 4670122.3 |  |
| mgm4670123.3 | 4670123.3 |  |
| mgm4679248.3 | 4679248.3 |  |
| mgm4679249.3 | 4679249.3 |  |
| mgm4679250.3 | 4679250.3 |  |
| mgm4679251.3 | 4679251.3 |  |
| mgm4679252.3 | 4679252.3 |  |
| mgm4679253.3 | 4679253.3 |  |
| mgm4679254.3 | 4679254.3 |  |
| mgm4679255.3 | 4679255.3 |  |
| mgm4679256.3 | 4679256.3 |  |
| mgm4679257.3 | 4679257.3 |  |
| mgm4679258.3 | 4679258.3 |  |
| mgm4679259.3 | 4679259.3 |  |
| mgm4679260.3 | 4679260.3 |  |
| mgm4679261.3 | 4679261.3 |  |
| mgm4679262.3 | 4679262.3 |  |
| mgm4679263.3 | 4679263.3 |  |
| mgm4679264.3 | 4679264.3 |  |
| mgm4679265.3 | 4679265.3 |  |
| mgm4679266.3 | 4679266.3 |  |
| mgm4679267.3 | 4679267.3 |  |
| mgm4679268.3 | 4679268.3 |  |
| mgm4679269.3 | 4679269.3 |  |
| mgm4679270.3 | 4679270.3 |  |
| mgm4679271.3 | 4679271.3 |  |
| mgm4679272.3 | 4679272.3 |  |
| mgm4679273.3 | 4679273.3 |  |
| mgm4679274.3 | 4679274.3 |  |
| mgm4679275.3 | 4679275.3 |  |
| mgm4679276.3 | 4679276.3 |  |
| mgm4679277.3 | 4679277.3 |  |
| mgm4679278.3 | 4679278.3 |  |
| mgm4679279.3 | 4679279.3 |  |
| mgm4679280.3 | 4679280.3 |  |
| mgm4679281.3 | 4679281.3 |  |
| mgm4679282.3 | 4679282.3 |  |
| mgm4679283.3 | 4679283.3 |  |
| mgm4679284.3 | 4679284.3 |  |
| mgm4679285.3 | 4679285.3 |  |
| mgm4679286.3 | 4679286.3 |  |
| mgm4679287.3 | 4679287.3 |  |
| mgm4679288.3 | 4679288.3 |  |
| mgm4679289.3 | 4679289.3 |  |
| mgm4679290.3 | 4679290.3 |  |
| mgm4679291.3 | 4679291.3 |  |
| mgm4679292.3 | 4679292.3 |  |
| mgm4683353.3 | 4683353.3 |  |
| mgm4683369.3 | 4683369.3 |  |
| mgm4683391.3 | 4683391.3 |  |
| mgm4683392.3 | 4683392.3 |  |
| mgm4683393.3 | 4683393.3 |  |
| mgm4683394.3 | 4683394.3 |  |
| mgm4683418.3 | 4683418.3 |  |
| mgm4683431.3 | 4683431.3 |  |
| mgm4684108.3 | 4684108.3 |  |
| mgm4684109.3 | 4684109.3 |  |
| mgm4688374.3 | 4688374.3 |  |
| mgm4689226.3 | 4689226.3 |  |
| mgm4689227.3 | 4689227.3 |  |
| mgm4689539.3 | 4689539.3 |  |
| mgm4690364.3 | 4690364.3 |  |
| mgm4690365.3 | 4690365.3 |  |
| mgm4690366.3 | 4690366.3 |  |
| mgm4690367.3 | 4690367.3 |  |
| mgm4690368.3 | 4690368.3 |  |
| mgm4690369.3 | 4690369.3 |  |
| mgm4690370.3 | 4690370.3 |  |
| mgm4690371.3 | 4690371.3 |  |
| mgm4690372.3 | 4690372.3 |  |
| mgm4690373.3 | 4690373.3 |  |
| mgm4690374.3 | 4690374.3 |  |
| mgm4690375.3 | 4690375.3 |  |
| mgm4690376.3 | 4690376.3 |  |
| mgm4690377.3 | 4690377.3 |  |
| mgm4690378.3 | 4690378.3 |  |
| mgm4690389.3 | 4690389.3 |  |
| mgm4690390.3 | 4690390.3 |  |
| mgm4690391.3 | 4690391.3 |  |
| mgm4690392.3 | 4690392.3 |  |
| mgm4690393.3 | 4690393.3 |  |
| mgm4690394.3 | 4690394.3 |  |
| mgm4690395.3 | 4690395.3 |  |
| mgm4690396.3 | 4690396.3 |  |
| mgm4690397.3 | 4690397.3 |  |
| mgm4690398.3 | 4690398.3 |  |
| mgm4690399.3 | 4690399.3 |  |
| mgm4690401.3 | 4690401.3 |  |
| mgm4690402.3 | 4690402.3 |  |
| mgm4690403.3 | 4690403.3 |  |
| mgm4690404.3 | 4690404.3 |  |
| mgm4690405.3 | 4690405.3 |  |
| mgm4690406.3 | 4690406.3 |  |
| mgm4690408.3 | 4690408.3 |  |
| mgm4690409.3 | 4690409.3 |  |
| mgm4690410.3 | 4690410.3 |  |
| mgm4690411.3 | 4690411.3 |  |
| mgm4690412.3 | 4690412.3 |  |
| mgm4690413.3 | 4690413.3 |  |
| mgm4690414.3 | 4690414.3 |  |
| mgm4690415.3 | 4690415.3 |  |
| mgm4690416.3 | 4690416.3 |  |
| mgm4690417.3 | 4690417.3 |  |
| mgm4690418.3 | 4690418.3 |  |
| mgm4690419.3 | 4690419.3 |  |
| mgm4690421.3 | 4690421.3 |  |
| mgm4690422.3 | 4690422.3 |  |
| mgm4690423.3 | 4690423.3 |  |
| mgm4690424.3 | 4690424.3 |  |
| mgm4690425.3 | 4690425.3 |  |
| mgm4690426.3 | 4690426.3 |  |
| mgm4690428.3 | 4690428.3 |  |
| mgm4690500.3 | 4690500.3 |  |
| mgm4690501.3 | 4690501.3 |  |
| mgm4690502.3 | 4690502.3 |  |
| mgm4690503.3 | 4690503.3 |  |
| mgm4690504.3 | 4690504.3 |  |
| mgm4690506.3 | 4690506.3 |  |
| mgm4690507.3 | 4690507.3 |  |
| mgm4690508.3 | 4690508.3 |  |
| mgm4690509.3 | 4690509.3 |  |
| mgm4690510.3 | 4690510.3 |  |
| mgm4690512.3 | 4690512.3 |  |
| mgm4690513.3 | 4690513.3 |  |
| mgm4690515.3 | 4690515.3 |  |
| mgm4690516.3 | 4690516.3 |  |
| mgm4690517.3 | 4690517.3 |  |
| mgm4690518.3 | 4690518.3 |  |
| mgm4690519.3 | 4690519.3 |  |
| mgm4690520.3 | 4690520.3 |  |
| mgm4690521.3 | 4690521.3 |  |
| mgm4690523.3 | 4690523.3 |  |
| mgm4690524.3 | 4690524.3 |  |
| mgm4690525.3 | 4690525.3 |  |
| mgm4690526.3 | 4690526.3 |  |
| mgm4690527.3 | 4690527.3 |  |
| mgm4690528.3 | 4690528.3 |  |
| mgm4690530.3 | 4690530.3 |  |
| mgm4690531.3 | 4690531.3 |  |
| mgm4690533.3 | 4690533.3 |  |
| mgm4690534.3 | 4690534.3 |  |
| mgm4690535.3 | 4690535.3 |  |
| mgm4690537.3 | 4690537.3 |  |
| mgm4690538.3 | 4690538.3 |  |
| mgm4690539.3 | 4690539.3 |  |
| mgm4690540.3 | 4690540.3 |  |
| mgm4690541.3 | 4690541.3 |  |
| mgm4690542.3 | 4690542.3 |  |
| mgm4690543.3 | 4690543.3 |  |
| mgm4690545.3 | 4690545.3 |  |
| mgm4690930.3 | 4690930.3 |  |
| mgm4690945.3 | 4690945.3 |  |
| mgm4690985.3 | 4690985.3 |  |
| mgm4690987.3 | 4690987.3 |  |
| mgm4690989.3 | 4690989.3 |  |
| mgm4691267.3 | 4691267.3 |  |
| mgm4692699.3 | 4692699.3 |  |
| mgm4692700.3 | 4692700.3 |  |
| mgm4692701.3 | 4692701.3 |  |
| mgm4692702.3 | 4692702.3 |  |
| mgm4692703.3 | 4692703.3 |  |
| mgm4692704.3 | 4692704.3 |  |
| mgm4692705.3 | 4692705.3 |  |
| mgm4692706.3 | 4692706.3 |  |
| mgm4692707.3 | 4692707.3 |  |
| mgm4692708.3 | 4692708.3 |  |
| mgm4692709.3 | 4692709.3 |  |
| mgm4692710.3 | 4692710.3 |  |
| mgm4692711.3 | 4692711.3 |  |
| mgm4692712.3 | 4692712.3 |  |
| mgm4692713.3 | 4692713.3 |  |
| mgm4693638.3 | 4693638.3 |  |
| mgm4694153.3 | 4694153.3 |  |
| mgm4694154.3 | 4694154.3 |  |
| mgm4694747.3 | 4694747.3 |  |
| mgm4696917.3 | 4696917.3 |  |
| mgm4697387.3 | 4697387.3 |  |
| mgm4697756.3 | 4697756.3 |  |
| mgm4697870.3 | 4697870.3 |  |
| mgm4697871.3 | 4697871.3 |  |
| mgm4697872.3 | 4697872.3 |  |
| mgm4697873.3 | 4697873.3 |  |
| mgm4697874.3 | 4697874.3 |  |
| mgm4697875.3 | 4697875.3 |  |
| mgm4697876.3 | 4697876.3 |  |
| mgm4697877.3 | 4697877.3 |  |
| mgm4697878.3 | 4697878.3 |  |
| mgm4697879.3 | 4697879.3 |  |
| mgm4697880.3 | 4697880.3 |  |
| mgm4697881.3 | 4697881.3 |  |
| mgm4697882.3 | 4697882.3 |  |
| mgm4697883.3 | 4697883.3 |  |
| mgm4697884.3 | 4697884.3 |  |
| mgm4698132.3 | 4698132.3 |  |
| mgm4702966.3 | 4702966.3 |  |
| mgm4702967.3 | 4702967.3 |  |
| mgm4702968.3 | 4702968.3 |  |
| mgm4702969.3 | 4702969.3 |  |
| mgm4702970.3 | 4702970.3 |  |
| mgm4702971.3 | 4702971.3 |  |
| mgm4702972.3 | 4702972.3 |  |
| mgm4702973.3 | 4702973.3 |  |
| mgm4702974.3 | 4702974.3 |  |
| mgm4702975.3 | 4702975.3 |  |
| mgm4702976.3 | 4702976.3 |  |
| mgm4702977.3 | 4702977.3 |  |
| mgm4702978.3 | 4702978.3 |  |
| mgm4702979.3 | 4702979.3 |  |
| mgm4702980.3 | 4702980.3 |  |
| mgm4702981.3 | 4702981.3 |  |
| mgm4702982.3 | 4702982.3 |  |
| mgm4702983.3 | 4702983.3 |  |
| mgm4702984.3 | 4702984.3 |  |
| mgm4702985.3 | 4702985.3 |  |
| mgm4702986.3 | 4702986.3 |  |
| mgm4702987.3 | 4702987.3 |  |
| mgm4702988.3 | 4702988.3 |  |
| mgm4702989.3 | 4702989.3 |  |
| mgm4702990.3 | 4702990.3 |  |
| mgm4702991.3 | 4702991.3 |  |
| mgm4702992.3 | 4702992.3 |  |
| mgm4702993.3 | 4702993.3 |  |
| mgm4702994.3 | 4702994.3 |  |
| mgm4702995.3 | 4702995.3 |  |
| mgm4704047.3 | 4704047.3 |  |
| mgm4704048.3 | 4704048.3 |  |
| mgm4704049.3 | 4704049.3 |  |
| mgm4704050.3 | 4704050.3 |  |
| mgm4704051.3 | 4704051.3 |  |
| mgm4704052.3 | 4704052.3 |  |
| mgm4704053.3 | 4704053.3 |  |
| mgm4704054.3 | 4704054.3 |  |
| mgm4704055.3 | 4704055.3 |  |
| mgm4704056.3 | 4704056.3 |  |
| mgm4704057.3 | 4704057.3 |  |
| mgm4704058.3 | 4704058.3 |  |
| mgm4704059.3 | 4704059.3 |  |
| mgm4704060.3 | 4704060.3 |  |
| mgm4704061.3 | 4704061.3 |  |
| mgm4704062.3 | 4704062.3 |  |
| mgm4705012.3 | 4705012.3 |  |
| mgm4705013.3 | 4705013.3 |  |
| mgm4705014.3 | 4705014.3 |  |
| mgm4705015.3 | 4705015.3 |  |
| mgm4705016.3 | 4705016.3 |  |
| mgm4705017.3 | 4705017.3 |  |
| mgm4705018.3 | 4705018.3 |  |
| mgm4705019.3 | 4705019.3 |  |
| mgm4705020.3 | 4705020.3 |  |
| mgm4705021.3 | 4705021.3 |  |
| mgm4705022.3 | 4705022.3 |  |
| mgm4705023.3 | 4705023.3 |  |
| mgm4705024.3 | 4705024.3 |  |
| mgm4705025.3 | 4705025.3 |  |
| mgm4705026.3 | 4705026.3 |  |
| mgm4705027.3 | 4705027.3 |  |
| mgm4705028.3 | 4705028.3 |  |
| mgm4705029.3 | 4705029.3 |  |
| mgm4705031.3 | 4705031.3 |  |
| mgm4705032.3 | 4705032.3 |  |
| mgm4705033.3 | 4705033.3 |  |
| mgm4705034.3 | 4705034.3 |  |
| mgm4705035.3 | 4705035.3 |  |
| mgm4705036.3 | 4705036.3 |  |
| mgm4705037.3 | 4705037.3 |  |
| mgm4705038.3 | 4705038.3 |  |
| mgm4707558.3 | 4707558.3 |  |
| mgm4707559.3 | 4707559.3 |  |
| mgm4707560.3 | 4707560.3 |  |
| mgm4707561.3 | 4707561.3 |  |
| mgm4707562.3 | 4707562.3 |  |
| mgm4707563.3 | 4707563.3 |  |
| mgm4707564.3 | 4707564.3 |  |
| mgm4707565.3 | 4707565.3 |  |
| mgm4707566.3 | 4707566.3 |  |
| mgm4707567.3 | 4707567.3 |  |
| mgm4707568.3 | 4707568.3 |  |
| mgm4707569.3 | 4707569.3 |  |
| mgm4707570.3 | 4707570.3 |  |
| mgm4707571.3 | 4707571.3 |  |
| mgm4707572.3 | 4707572.3 |  |
| mgm4707573.3 | 4707573.3 |  |
| mgm4707574.3 | 4707574.3 |  |
| mgm4707575.3 | 4707575.3 |  |
| mgm4707576.3 | 4707576.3 |  |
| mgm4707577.3 | 4707577.3 |  |
| mgm4707578.3 | 4707578.3 |  |
| mgm4707579.3 | 4707579.3 |  |
| mgm4707580.3 | 4707580.3 |  |
| mgm4707581.3 | 4707581.3 |  |
| mgm4707582.3 | 4707582.3 |  |
| mgm4707583.3 | 4707583.3 |  |
| mgm4707584.3 | 4707584.3 |  |
| mgm4707585.3 | 4707585.3 |  |
| mgm4707586.3 | 4707586.3 |  |
| mgm4707587.3 | 4707587.3 |  |
| mgm4707588.3 | 4707588.3 |  |
| mgm4707589.3 | 4707589.3 |  |
| mgm4712230.3 | 4712230.3 |  |
| mgm4712232.3 | 4712232.3 |  |
| mgm4712233.3 | 4712233.3 |  |
| mgm4712234.3 | 4712234.3 |  |
| mgm4712235.3 | 4712235.3 |  |
| mgm4712236.3 | 4712236.3 |  |
| mgm4712237.3 | 4712237.3 |  |
| mgm4712238.3 | 4712238.3 |  |
| mgm4712239.3 | 4712239.3 |  |
| mgm4712241.3 | 4712241.3 |  |
| mgm4712242.3 | 4712242.3 |  |
| mgm4712243.3 | 4712243.3 |  |
| mgm4712245.3 | 4712245.3 |  |
| mgm4712246.3 | 4712246.3 |  |
| mgm4712247.3 | 4712247.3 |  |
| mgm4712248.3 | 4712248.3 |  |
| mgm4712249.3 | 4712249.3 |  |
| mgm4712250.3 | 4712250.3 |  |
| mgm4712251.3 | 4712251.3 |  |
| mgm4712252.3 | 4712252.3 |  |
| mgm4712253.3 | 4712253.3 |  |
| mgm4712254.3 | 4712254.3 |  |
| mgm4712255.3 | 4712255.3 |  |
| mgm4712256.3 | 4712256.3 |  |
| mgm4712257.3 | 4712257.3 |  |
| mgm4712258.3 | 4712258.3 |  |
| mgm4712259.3 | 4712259.3 |  |
| mgm4712260.3 | 4712260.3 |  |
| mgm4712261.3 | 4712261.3 |  |
| mgm4712262.3 | 4712262.3 |  |
| mgm4712263.3 | 4712263.3 |  |
| mgm4712264.3 | 4712264.3 |  |
| mgm4712265.3 | 4712265.3 |  |
| mgm4712266.3 | 4712266.3 |  |
| mgm4712267.3 | 4712267.3 |  |
| mgm4712268.3 | 4712268.3 |  |
| mgm4712270.3 | 4712270.3 |  |
| mgm4712271.3 | 4712271.3 |  |
| mgm4712272.3 | 4712272.3 |  |
| mgm4712273.3 | 4712273.3 |  |
| mgm4717306.3 | 4717306.3 |  |
| mgm4717307.3 | 4717307.3 |  |
| mgm4717308.3 | 4717308.3 |  |
| mgm4717309.3 | 4717309.3 |  |
| mgm4717310.3 | 4717310.3 |  |
| mgm4717311.3 | 4717311.3 |  |
| mgm4717312.3 | 4717312.3 |  |
| mgm4717313.3 | 4717313.3 |  |
| mgm4717314.3 | 4717314.3 |  |
| mgm4717315.3 | 4717315.3 |  |
| mgm4717316.3 | 4717316.3 |  |
| mgm4717317.3 | 4717317.3 |  |
| mgm4717318.3 | 4717318.3 |  |
| mgm4717319.3 | 4717319.3 |  |
| mgm4717320.3 | 4717320.3 |  |
| mgm4717321.3 | 4717321.3 |  |
| mgm4717322.3 | 4717322.3 |  |
| mgm4717323.3 | 4717323.3 |  |
| mgm4717324.3 | 4717324.3 |  |
| mgm4717325.3 | 4717325.3 |  |
| mgm4717326.3 | 4717326.3 |  |
| mgm4717327.3 | 4717327.3 |  |
| mgm4717328.3 | 4717328.3 |  |
| mgm4717329.3 | 4717329.3 |  |
| mgm4717330.3 | 4717330.3 |  |
| mgm4717331.3 | 4717331.3 |  |
| mgm4717332.3 | 4717332.3 |  |
| mgm4717333.3 | 4717333.3 |  |
| mgm4717334.3 | 4717334.3 |  |
| mgm4717335.3 | 4717335.3 |  |
| mgm4717336.3 | 4717336.3 |  |
| mgm4717337.3 | 4717337.3 |  |
| mgm4717338.3 | 4717338.3 |  |
| mgm4717339.3 | 4717339.3 |  |
| mgm4717340.3 | 4717340.3 |  |
| mgm4717341.3 | 4717341.3 |  |
| mgm4717342.3 | 4717342.3 |  |
| mgm4717343.3 | 4717343.3 |  |
| mgm4717344.3 | 4717344.3 |  |
| mgm4717345.3 | 4717345.3 |  |
| mgm4717346.3 | 4717346.3 |  |
| mgm4717347.3 | 4717347.3 |  |
| mgm4717348.3 | 4717348.3 |  |
| mgm4717349.3 | 4717349.3 |  |
| mgm4717350.3 | 4717350.3 |  |
| mgm4717351.3 | 4717351.3 |  |
| mgm4717378.3 | 4717378.3 |  |
| mgm4721484.3 | 4721484.3 |  |
| mgm4721485.3 | 4721485.3 |  |
| mgm4721486.3 | 4721486.3 |  |
| mgm4721487.3 | 4721487.3 |  |
| mgm4721488.3 | 4721488.3 |  |
| mgm4721489.3 | 4721489.3 |  |
| mgm4721490.3 | 4721490.3 |  |
| mgm4721491.3 | 4721491.3 |  |
| mgm4721492.3 | 4721492.3 |  |
| mgm4721493.3 | 4721493.3 |  |
| mgm4721494.3 | 4721494.3 |  |
| mgm4721495.3 | 4721495.3 |  |
| mgm4721496.3 | 4721496.3 |  |
| mgm4721497.3 | 4721497.3 |  |
| mgm4721498.3 | 4721498.3 |  |
| mgm4721499.3 | 4721499.3 |  |
| mgm4721500.3 | 4721500.3 |  |
| mgm4721501.3 | 4721501.3 |  |
| mgm4721502.3 | 4721502.3 |  |
| mgm4721503.3 | 4721503.3 |  |
| mgm4721504.3 | 4721504.3 |  |
| mgm4721505.3 | 4721505.3 |  |
| mgm4721506.3 | 4721506.3 |  |
| mgm4721507.3 | 4721507.3 |  |
| mgm4722870.3 | 4722870.3 |  |
| mgm4722871.3 | 4722871.3 |  |
| mgm4722872.3 | 4722872.3 |  |
| mgm4722873.3 | 4722873.3 |  |
| mgm4722874.3 | 4722874.3 |  |
| mgm4722875.3 | 4722875.3 |  |
| mgm4722876.3 | 4722876.3 |  |
| mgm4722877.3 | 4722877.3 |  |
| mgm4722878.3 | 4722878.3 |  |
| mgm4722879.3 | 4722879.3 |  |
| mgm4722880.3 | 4722880.3 |  |
| mgm4722881.3 | 4722881.3 |  |
| mgm4722882.3 | 4722882.3 |  |
| mgm4722883.3 | 4722883.3 |  |
| mgm4722884.3 | 4722884.3 |  |
| mgm4722885.3 | 4722885.3 |  |
| mgm4723157.3 | 4723157.3 |  |
| mgm4723160.3 | 4723160.3 |  |
| mgm4723162.3 | 4723162.3 |  |
| mgm4723166.3 | 4723166.3 |  |
| mgm4723167.3 | 4723167.3 |  |
| mgm4723168.3 | 4723168.3 |  |
| mgm4723169.3 | 4723169.3 |  |
| mgm4723170.3 | 4723170.3 |  |
| mgm4723171.3 | 4723171.3 |  |
| mgm4723172.3 | 4723172.3 |  |
| mgm4723173.3 | 4723173.3 |  |
| mgm4723174.3 | 4723174.3 |  |
| mgm4723175.3 | 4723175.3 |  |
| mgm4723176.3 | 4723176.3 |  |
| mgm4723177.3 | 4723177.3 |  |
| mgm4723178.3 | 4723178.3 |  |
| mgm4723179.3 | 4723179.3 |  |
| mgm4727373.3 | 4727373.3 |  |
| mgm4727374.3 | 4727374.3 |  |
| mgm4727375.3 | 4727375.3 |  |
| mgm4727376.3 | 4727376.3 |  |
| mgm4727377.3 | 4727377.3 |  |
| mgm4727378.3 | 4727378.3 |  |
| mgm4727379.3 | 4727379.3 |  |
| mgm4727380.3 | 4727380.3 |  |
| mgm4727381.3 | 4727381.3 |  |
| mgm4727382.3 | 4727382.3 |  |
| mgm4727383.3 | 4727383.3 |  |
| mgm4727384.3 | 4727384.3 |  |
| mgm4727385.3 | 4727385.3 |  |
| mgm4727386.3 | 4727386.3 |  |
| mgm4727387.3 | 4727387.3 |  |
| mgm4727388.3 | 4727388.3 |  |
| mgm4727389.3 | 4727389.3 |  |
| mgm4727390.3 | 4727390.3 |  |
| mgm4727391.3 | 4727391.3 |  |
| mgm4727392.3 | 4727392.3 |  |
| mgm4727393.3 | 4727393.3 |  |
| mgm4727394.3 | 4727394.3 |  |
| mgm4727395.3 | 4727395.3 |  |
| mgm4727396.3 | 4727396.3 |  |
| mgm4728762.3 | 4728762.3 |  |
| mgm4728763.3 | 4728763.3 |  |
| mgm4728764.3 | 4728764.3 |  |
| mgm4728765.3 | 4728765.3 |  |
| mgm4728766.3 | 4728766.3 |  |
| mgm4728767.3 | 4728767.3 |  |
| mgm4728768.3 | 4728768.3 |  |
| mgm4728769.3 | 4728769.3 |  |
| mgm4728770.3 | 4728770.3 |  |
| mgm4728771.3 | 4728771.3 |  |
| mgm4728772.3 | 4728772.3 |  |
| mgm4728773.3 | 4728773.3 |  |
| mgm4728774.3 | 4728774.3 |  |
| mgm4728775.3 | 4728775.3 |  |
| mgm4728776.3 | 4728776.3 |  |
| mgm4728777.3 | 4728777.3 |  |
| mgm4728778.3 | 4728778.3 |  |
| mgm4728779.3 | 4728779.3 |  |
| mgm4728780.3 | 4728780.3 |  |
| mgm4728781.3 | 4728781.3 |  |
| mgm4728782.3 | 4728782.3 |  |
| mgm4728783.3 | 4728783.3 |  |
| mgm4728784.3 | 4728784.3 |  |
| mgm4729129.3 | 4729129.3 |  |
| mgm4729130.3 | 4729130.3 |  |
| mgm4729131.3 | 4729131.3 |  |
| mgm4729132.3 | 4729132.3 |  |
| mgm4729133.3 | 4729133.3 |  |
| mgm4729134.3 | 4729134.3 |  |
| mgm4729135.3 | 4729135.3 |  |
| mgm4729136.3 | 4729136.3 |  |
| mgm4729137.3 | 4729137.3 |  |
| mgm4729138.3 | 4729138.3 |  |
| mgm4729140.3 | 4729140.3 |  |
| mgm4729141.3 | 4729141.3 |  |
| mgm4729142.3 | 4729142.3 |  |
| mgm4729143.3 | 4729143.3 |  |
| mgm4729144.3 | 4729144.3 |  |
| mgm4729145.3 | 4729145.3 |  |
| mgm4729146.3 | 4729146.3 |  |
| mgm4729147.3 | 4729147.3 |  |
| mgm4729148.3 | 4729148.3 |  |
| mgm4729149.3 | 4729149.3 |  |
| mgm4729150.3 | 4729150.3 |  |
| mgm4729151.3 | 4729151.3 |  |
| mgm4729152.3 | 4729152.3 |  |
| mgm4729773.3 | 4729773.3 |  |
| mgm4729774.3 | 4729774.3 |  |
| mgm4729775.3 | 4729775.3 |  |
| mgm4729776.3 | 4729776.3 |  |
| mgm4729777.3 | 4729777.3 |  |
| mgm4729778.3 | 4729778.3 |  |
| mgm4729779.3 | 4729779.3 |  |
| mgm4729780.3 | 4729780.3 |  |
| mgm4729781.3 | 4729781.3 |  |
| mgm4729782.3 | 4729782.3 |  |
| mgm4729783.3 | 4729783.3 |  |
| mgm4729784.3 | 4729784.3 |  |
| mgm4729903.3 | 4729903.3 |  |
| mgm4730672.3 | 4730672.3 |  |
| mgm4730673.3 | 4730673.3 |  |
| mgm4730674.3 | 4730674.3 |  |
| mgm4730675.3 | 4730675.3 |  |
| mgm4730676.3 | 4730676.3 |  |
| mgm4730677.3 | 4730677.3 |  |
| mgm4730678.3 | 4730678.3 |  |
| mgm4730679.3 | 4730679.3 |  |
| mgm4730844.3 | 4730844.3 |  |
| mgm4730845.3 | 4730845.3 |  |
| mgm4730847.3 | 4730847.3 |  |
| mgm4735485.3 | 4735485.3 |  |
| mgm4735486.3 | 4735486.3 |  |
| mgm4735487.3 | 4735487.3 |  |
| mgm4735489.3 | 4735489.3 |  |
| mgm4741982.3 | 4741982.3 |  |
| mgm4741983.3 | 4741983.3 |  |
| mgm4742537.3 | 4742537.3 |  |
| mgm4742538.3 | 4742538.3 |  |
| mgm4742540.3 | 4742540.3 |  |
| mgm4742545.3 | 4742545.3 |  |
| mgm4743979.3 | 4743979.3 |  |
| mgm4743980.3 | 4743980.3 |  |
| mgm4749373.3 | 4749373.3 |  |
| mgm4749374.3 | 4749374.3 |  |
| mgm4749375.3 | 4749375.3 |  |
| mgm4749376.3 | 4749376.3 |  |
| mgm4749377.3 | 4749377.3 |  |
| mgm4750361.3 | 4750361.3 |  |
| mgm4750362.3 | 4750362.3 |  |
| mgm4750363.3 | 4750363.3 |  |
| mgm4750364.3 | 4750364.3 |  |
| mgm4750365.3 | 4750365.3 |  |
| mgm4750366.3 | 4750366.3 |  |
| mgm4750367.3 | 4750367.3 |  |
| mgm4750368.3 | 4750368.3 |  |
| mgm4750369.3 | 4750369.3 |  |
| mgm4750370.3 | 4750370.3 |  |
| mgm4750371.3 | 4750371.3 |  |
| mgm4750372.3 | 4750372.3 |  |
| mgm4750373.3 | 4750373.3 |  |
| mgm4750374.3 | 4750374.3 |  |
| mgm4750375.3 | 4750375.3 |  |
| mgm4750376.3 | 4750376.3 |  |
| mgm4750377.3 | 4750377.3 |  |
| mgm4750378.3 | 4750378.3 |  |
| mgm4750379.3 | 4750379.3 |  |
| mgm4750380.3 | 4750380.3 |  |
| mgm4750381.3 | 4750381.3 |  |
| mgm4750382.3 | 4750382.3 |  |
| mgm4750383.3 | 4750383.3 |  |
| mgm4750384.3 | 4750384.3 |  |
| mgm4750385.3 | 4750385.3 |  |
| mgm4750386.3 | 4750386.3 |  |
| mgm4750387.3 | 4750387.3 |  |
| mgm4750388.3 | 4750388.3 |  |
| mgm4750389.3 | 4750389.3 |  |
| mgm4750390.3 | 4750390.3 |  |
| mgm4750391.3 | 4750391.3 |  |
| mgm4750392.3 | 4750392.3 |  |
| mgm4750393.3 | 4750393.3 |  |
| mgm4750394.3 | 4750394.3 |  |
| mgm4750395.3 | 4750395.3 |  |
| mgm4750396.3 | 4750396.3 |  |
| mgm4750397.3 | 4750397.3 |  |
| mgm4750398.3 | 4750398.3 |  |
| mgm4750399.3 | 4750399.3 |  |
| mgm4750400.3 | 4750400.3 |  |
| mgm4750401.3 | 4750401.3 |  |
| mgm4750402.3 | 4750402.3 |  |
| mgm4750403.3 | 4750403.3 |  |
| mgm4750404.3 | 4750404.3 |  |
| mgm4750405.3 | 4750405.3 |  |
| mgm4750406.3 | 4750406.3 |  |
| mgm4750407.3 | 4750407.3 |  |
| mgm4750408.3 | 4750408.3 |  |
| mgm4750409.3 | 4750409.3 |  |
| mgm4750410.3 | 4750410.3 |  |
| mgm4767414.3 | 4767414.3 |  |
| mgm4767415.3 | 4767415.3 |  |
| mgm4767417.3 | 4767417.3 |  |
| mgm4767418.3 | 4767418.3 |  |
| mgm4767419.3 | 4767419.3 |  |
| mgm4767420.3 | 4767420.3 |  |
| mgm4767421.3 | 4767421.3 |  |
| mgm4767422.3 | 4767422.3 |  |
| mgm4767423.3 | 4767423.3 |  |
| mgm4767424.3 | 4767424.3 |  |
| mgm4767425.3 | 4767425.3 |  |
| mgm4767426.3 | 4767426.3 |  |
| mgm4767427.3 | 4767427.3 |  |
| mgm4767428.3 | 4767428.3 |  |
| mgm4767429.3 | 4767429.3 |  |
| mgm4767430.3 | 4767430.3 |  |
| mgm4767431.3 | 4767431.3 |  |
| mgm4767432.3 | 4767432.3 |  |
| mgm4767433.3 | 4767433.3 |  |
| mgm4767434.3 | 4767434.3 |  |
| mgm4767435.3 | 4767435.3 |  |
| mgm4767436.3 | 4767436.3 |  |
| mgm4767437.3 | 4767437.3 |  |
| mgm4767438.3 | 4767438.3 |  |
| mgm4767439.3 | 4767439.3 |  |
| mgm4767440.3 | 4767440.3 |  |
| mgm4767441.3 | 4767441.3 |  |
| mgm4767442.3 | 4767442.3 |  |
| mgm4767443.3 | 4767443.3 |  |
| mgm4767444.3 | 4767444.3 |  |
| mgm4767445.3 | 4767445.3 |  |
| mgm4767446.3 | 4767446.3 |  |
| mgm4767447.3 | 4767447.3 |  |
| mgm4767448.3 | 4767448.3 |  |
| mgm4767449.3 | 4767449.3 |  |
| mgm4767450.3 | 4767450.3 |  |
| mgm4767451.3 | 4767451.3 |  |
| mgm4767452.3 | 4767452.3 |  |
| mgm4767453.3 | 4767453.3 |  |
| mgm4767454.3 | 4767454.3 |  |
| mgm4767455.3 | 4767455.3 |  |
| mgm4767456.3 | 4767456.3 |  |
| mgm4767457.3 | 4767457.3 |  |
| mgm4767458.3 | 4767458.3 |  |
| mgm4767459.3 | 4767459.3 |  |
| mgm4767460.3 | 4767460.3 |  |
| mgm4767461.3 | 4767461.3 |  |
| mgm4767462.3 | 4767462.3 |  |
| mgm4767463.3 | 4767463.3 |  |
| mgm4767464.3 | 4767464.3 |  |
| mgm4767465.3 | 4767465.3 |  |
| mgm4767466.3 | 4767466.3 |  |
| mgm4767467.3 | 4767467.3 |  |
| mgm4767468.3 | 4767468.3 |  |
| mgm4767469.3 | 4767469.3 |  |
| mgm4767470.3 | 4767470.3 |  |
| mgm4767471.3 | 4767471.3 |  |
| mgm4767472.3 | 4767472.3 |  |
| mgm4767473.3 | 4767473.3 |  |
| mgm4767474.3 | 4767474.3 |  |
| mgm4767475.3 | 4767475.3 |  |
| mgm4767476.3 | 4767476.3 |  |
| mgm4767477.3 | 4767477.3 |  |
| mgm4767478.3 | 4767478.3 |  |
| mgm4767479.3 | 4767479.3 |  |
| mgm4767480.3 | 4767480.3 |  |
| mgm4767481.3 | 4767481.3 |  |
| mgm4767483.3 | 4767483.3 |  |
| mgm4767484.3 | 4767484.3 |  |
| mgm4767485.3 | 4767485.3 |  |
| mgm4767486.3 | 4767486.3 |  |
| mgm4767487.3 | 4767487.3 |  |
| mgm4767488.3 | 4767488.3 |  |
| mgm4767489.3 | 4767489.3 |  |
| mgm4767490.3 | 4767490.3 |  |
| mgm4767491.3 | 4767491.3 |  |
| mgm4767492.3 | 4767492.3 |  |
| mgm4767493.3 | 4767493.3 |  |
| mgm4767494.3 | 4767494.3 |  |
| mgm4767495.3 | 4767495.3 |  |
| mgm4767496.3 | 4767496.3 |  |
| mgm4767497.3 | 4767497.3 |  |
| mgm4767498.3 | 4767498.3 |  |
| mgm4767499.3 | 4767499.3 |  |
| mgm4767500.3 | 4767500.3 |  |
| mgm4767501.3 | 4767501.3 |  |
| mgm4767502.3 | 4767502.3 |  |
| mgm4767503.3 | 4767503.3 |  |
| mgm4767504.3 | 4767504.3 |  |
| mgm4767505.3 | 4767505.3 |  |
| mgm4767506.3 | 4767506.3 |  |
| mgm4767507.3 | 4767507.3 |  |
| mgm4767508.3 | 4767508.3 |  |
| mgm4767509.3 | 4767509.3 |  |
| mgm4767510.3 | 4767510.3 |  |
| mgm4767511.3 | 4767511.3 |  |
| mgm4767512.3 | 4767512.3 |  |
| mgm4767513.3 | 4767513.3 |  |
| mgm4767514.3 | 4767514.3 |  |
| mgm4767515.3 | 4767515.3 |  |
| mgm4767516.3 | 4767516.3 |  |
| mgm4767517.3 | 4767517.3 |  |
| mgm4767518.3 | 4767518.3 |  |
| mgm4767519.3 | 4767519.3 |  |
| mgm4767520.3 | 4767520.3 |  |
| mgm4767521.3 | 4767521.3 |  |
| mgm4767522.3 | 4767522.3 |  |
| mgm4767523.3 | 4767523.3 |  |
| 1006_KanasCultivatedCorn |  | 1077705 |
| 1028_WisconsinContinuousCorn |  | 1077702 |
| 1028_WisconsinNativePrairie |  | 1077701 |
| 1209_IowaContinuousCorn |  | 1077703 |
| 1222_WisconsinContinuousCorn |  | 1077702 |
| 1224_WisconsinContinuousCorn |  | 1077702 |
| 1235_WisconsinNativePrairie |  | 1077701 |
| 1237_WisconsinNativePrairie |  | 1077701 |
| 1246_KanasCultivatedCorn |  | 1077705 |
| 1252_KanasCultivatedCorn |  | 1077705 |
| 1256_KanasNativePrairie |  | 1077706 |
| 1269_KanasNativePrairie |  | 1077706 |
| 1300_IowaContinuousCorn |  | 1077703 |
| 1300_KanasCultivatedCorn |  | 1077705 |
| 1311_WisconsinSwitchGrass |  | 1077700 |
| 1323_WisconsinRestoredPrairie |  | 1077699 |
| 1418_WisconsinSwitchGrass |  | 1077700 |
| 1424_IowaContinuousCorn |  | 1077703 |
| 1425_IowaContinuousCorn |  | 1077703 |
| 1427_IowaNativePrairie |  | 1077704 |
| 1461_IowaNativePrairie |  | 1077704 |
| 1477_WisconsinRestoredPrairie |  | 1077699 |
| 1508_WisconsinRestoredPrairie |  | 1077699 |
| 1559_WisconsinRestoredPrairie |  | 1077699 |
| 1651_KanasNativePrairie |  | 1077706 |
| 1652_KanasNativePrairie |  | 1077706 |
| 1653_KanasNativePrairie |  | 1077706 |
| 1654_KanasNativePrairie |  | 1077706 |
| 1658_KanasNativePrairie |  | 1077706 |
| 625_WisconsinRestoredPrairie |  | 1077699 |
| 649_IowaContinuousCorn |  | 1077703 |
| 723_WisconsinNativePrairie |  | 1077701 |
| 850_IowaNativePrairie |  | 1077704 |
| 868_IowaContinuousCorn |  | 1077703 |
| 895_IowaContinuousCorn |  | 1077703 |
| 895_IowaNativePrairie |  | 1077704 |
| 980_KanasNativePrairie |  | 1077706 |
