# Supplementary material for: MetaFunPrimer: an Environment-Specific, High-Throughput Primer Design Tool for Improved Quantification of Target Genes
Source: mSystems. 2021 Sep 21;6(5):e00201-21. doi: 10.1128/mSystems.00201-21 (PMC8547451; doi:10.1128/mSystems.00201-21)
Supplement: TABLE S2 [file msystems.00201-21-st002.docx]

**TABLE S2** Target *amo*A-AOB genes identified and ranked in 1,550 soil metagenomes. Representative genes originate from known *amo*A-AOB genes clustered at 96% amino acid sequence similarity. Representative score (R-score) is an average-weighted measurement of representative genes abundance and prevalence in soil metagenomes (see Materials and Methods section for details).

| Representative gene | Number of samples observed in | Number of reads associated with representative gene | R-Score | Cumulative  R-Score |
| --- | --- | --- | --- | --- |
| AAB38709 | 216 | 2381 | 0.99769 | 0.177 |
| ABM54175 | 217 | 1003 | 0.71050 | 0.303 |
| SEF68642 | 213 | 812 | 0.66112 | 0.421 |
| AAC25057 | 201 | 863 | 0.64406 | 0.536 |
| KIO48008 | 148 | 751 | 0.49784 | 0.624 |
| AFL48355 | 114 | 294 | 0.32313 | 0.682 |
| ADZ75349 | 119 | 238 | 0.32294 | 0.739 |
| AAL86638 | 54 | 105 | 0.14453 | 0.765 |
| AAL86637 | 51 | 114 | 0.13948 | 0.790 |
| ADZ75355 | 38 | 110 | 0.10855 | 0.809 |
| ABN04023 | 33 | 58 | 0.08605 | 0.824 |
| CAD62102 | 28 | 91 | 0.08141 | 0.839 |
| AFL48315 | 22 | 143 | 0.07844 | 0.853 |
| CAD62138 | 26 | 46 | 0.06732 | 0.865 |
| ABN12978 | 26 | 36 | 0.06522 | 0.876 |
| AFL48350 | 25 | 44 | 0.06459 | 0.888 |
| AAB38710 | 21 | 54 | 0.05743 | 0.898 |
| CAD62133 | 22 | 25 | 0.05365 | 0.908 |
| BAA92239 | 19 | 45 | 0.05091 | 0.917 |
| CAD62076 | 20 | 25 | 0.04902 | 0.925 |
| AEJ02636 | 20 | 24 | 0.04881 | 0.934 |
| AAG60667 | 20 | 24 | 0.04881 | 0.943 |
| ABB69924 | 11 | 114 | 0.04689 | 0.951 |
| ABN13016 | 12 | 15 | 0.02840 | 0.956 |
| ABR29000 | 12 | 14 | 0.02819 | 0.961 |
| AFI41714 | 7 | 54 | 0.02502 | 0.966 |
| ABN13073 | 10 | 10 | 0.02272 | 0.970 |
| AAO60366 | 7 | 38 | 0.02166 | 0.974 |
| ABM91864 | 9 | 12 | 0.02083 | 0.977 |
| ABN13100 | 9 | 9 | 0.02020 | 0.981 |
| AFL48320 | 7 | 7 | 0.01515 | 0.984 |
| ABN13071 | 6 | 10 | 0.01346 | 0.986 |
| ABN11248 | 6 | 10 | 0.01346 | 0.988 |
| CAD62091 | 5 | 8 | 0.01073 | 0.990 |
| CAD62077 | 4 | 4 | 0.00757 | 0.992 |
| ABN04006 | 4 | 4 | 0.00757 | 0.993 |
| AFL48358 | 3 | 4 | 0.00526 | 0.994 |
| AFL48409 | 3 | 3 | 0.00505 | 0.995 |
| AAC31361 | 3 | 3 | 0.00505 | 0.996 |
| AFI41578 | 2 | 5 | 0.00316 | 0.996 |
| AFL48391 | 2 | 3 | 0.00273 | 0.997 |
| ACY38032 | 2 | 3 | 0.00273 | 0.997 |
| CAD62415 | 2 | 2 | 0.00252 | 0.998 |
| CAD62411 | 2 | 2 | 0.00252 | 0.998 |
| AFI41653 | 2 | 2 | 0.00252 | 0.999 |
| ABN13004 | 2 | 2 | 0.00252 | 0.999 |
| ABN12996 | 2 | 2 | 0.00252 | 0.999 |
| BAD15293 | 1 | 2 | 0.00021 | 1.000 |
| ABV49213 | 1 | 1 | 0.00000 | 1.000 |
| AAF03938 | 1 | 1 | 0.00000 | 1.000 |
