# Supplementary material for: MetaFunPrimer: an Environment-Specific, High-Throughput Primer Design Tool for Improved Quantification of Target Genes
Source: mSystems. 2021 Sep 21;6(5):e00201-21. doi: 10.1128/mSystems.00201-21 (PMC8547451; doi:10.1128/mSystems.00201-21)
Supplement: TABLE S3 [file msystems.00201-21-st003.docx]

**TABLE S3** Primers were designed for 720 abundant *amo*A-AOB genes in 1,550 soil metagenomes through EcoFunPrimer, which is embedded in MetaFunPrimer. By allowing at most six degeneracies for each primer, 20 degenerate and 8 non-degenerate primer pairs were generated. Please note that the method shown in this paper is an example and users can generate all non-degenerate primers if needed. (https://github.com/rdpstaff/EcoFunPrimer).

| **Primer ID & sequence of degenerate primer** | **Primer sequences generated from EcoFunPrimer** |
| --- | --- |
| F:soilAbundant_amoA_AOB.C001  TTYGGHYTBYTGTTCTACCCKG | TTCGGTTTGTTGTTCTACCCTGG |
|  | TTTGGCCTGTTGTTCTACCCTG |
|  | TTTGGACTGTTGTTCTACCCTGG |
|  | TTTGGACTGTTGTTCTACCCGG |
|  | TTTGGTCTTCTGTTCTACCCGG |
|  | TTTGGACTCTTGTTCTACCCGG |
| R:soilAbundant_amoA_AOB.C001  TAGTAGAASGCGGTGCAGTAGA | TAGTAGAACGCGGTGCAGTAGA |
|  | TAGTAGAAGGCGGTGCAGTAGA |
| F:soilAbundant_amoA_AOB.C002  GACTACACCGGCTTYCTGTATG | GACTACACCGGCTTCCTGTATG |
|  | GACTACACCGGCTTTCTGTATGTAC |
| R:soilAbundant_amoA_AOB.C002  GTVACGTCGTTCTKCATGSWRA | GTGACGTCGTTCTTCATGCTGA |
|  | GTGACGTCGTTCTTCATGGTAAC |
|  | GTAACGTCGTTCTTCATGGTGAC |
|  | GTCACGTCGTTCTTCATGGAGA |
|  | GTAACGTCGTTCTGCATGGAGA |
| F:soilAbundant_amoA_AOB.C003  YTTCTACTGGTGGTCGCACTAC | TTTCTACTGGTGGTCGCACTAC |
|  | CTTCTACTGGTGGTCGCACTAC |
| R:soilAbundant_amoA_AOB.C003  GTTCGATSWKVCGYACGTAYTC | GTTCGATCTGACGCACGTACTC |
|  | GTTCGATGTTGCGTACGTACTC |
|  | GTTCGATCAGCCGTACGTATTCC |
|  | GTTCGATCAGACGCACGTACTC |
|  | GTTCGATCAGCCGTACGTACTC |
| F:soilAbundant_amoA_AOB.C004  ACTTCTGGCTBGACTGGAAAGA | ACTTCTGGCTCGACTGGAAAGA |
|  | ACTTCTGGCTTGACTGGAAAGA |
|  | ACTTCTGGCTGGACTGGAAAGA |
| R:soilAbundant_amoA_AOB.C004  RRTATCATGGTGGAGGGMAABA | GGTATCATGGTGGAGGGAAATACG |
|  | GGTATCATGGTGGAGGGCAATA |
|  | AATATCATGGTGGAGGGCAAGAC |
|  | GGTATCATGGTGGAGGGAAACA |
| F:soilAbundant_amoA_AOB.C005  GCBRCSWTSATGTAYTAYYTGTG | GCCACCATCATGTACTACCTGTG |
|  | GCTGCGATCATGTACTACCTGTG |
|  | GCGGCGTTGATGTATTATTTGTGG |
|  | GCTGCCATCATGTACTACCTGTG |
| R:soilAbundant_amoA_AOB.C005  YARDGCYGTDAYCAKCCAGTTR | TAATGCCGTTACCAGCCAGTTAC |
|  | CAGTGCCGTGATCATCCAGTTA |
|  | CAGAGCCGTGATCATCCAGTTG |
|  | CAGTGCTGTGATCATCCAGTTG |
|  | CAGGGCTGTAACCATCCAGTTG |
| F:soilAbundant_amoA_AOB.C006  RGCTTYCTGTATGTHCGYACSG | AGCTTTCTGTATGTACGCACGG |
|  | GGCTTTCTGTATGTCCGTACCG |
|  | GGCTTCCTGTATGTACGTACCG |
|  | GGCTTCCTGTATGTTCGTACCG |
| R:soilAbundant_amoA_AOB.C006  TMACGTCGTTCTKCATGSWGAY | TCACGTCGTTCTTCATGCTGAC |
|  | TAACGTCGTTCTGCATGGAGATG |
| F:soilAbundant_amoA_AOB.C007  GGGAYTTCTGGMTKGACTGGAA | GGGACTTCTGGATTGACTGGAAAG |
|  | GGGATTTCTGGATGGACTGGAA |
|  | GGGATTTCTGGCTTGACTGGAA |
| R:soilAbundant_amoA_AOB.C007  GGTATCATGGTGGAGGGMARYA | GGTATCATGGTGGAGGGCAGTA |
|  | GGTATCATGGTGGAGGGAAATACG |
|  | GGTATCATGGTGGAGGGCAATA |
|  | GGTATCATGGTGGAGGGAAACA |
| F:soilAbundant_amoA_AOB.C008  CCSATCAACTTYGTMYTKCCMTC | CCCATCAACTTCGTATTTCCCTCC |
|  | CCCATCAACTTCGTCTTTCCCTC |
|  | CCGATCAACTTTGTACTGCCATC |
| R:soilAbundant_amoA_AOB.C008  YTCRATCAGSCKHACRTAYTCM | TTCGATCAGCCGTACATACTCAG |
|  | TTCGATCAGGCGCACATATTCAG |
|  | TTCGATCAGCCTCACGTACTCA |
|  | CTCAATCAGGCGAACATATTCCG |
| F:soilAbundant_amoA_AOB.C009  GAYTACACCGGCTTCCTGTATG | GATTACACCGGCTTCCTGTATGTAC |
|  | GACTACACCGGCTTCCTGTATG |
| R:soilAbundant_amoA_AOB.C009  AATGCMGTSACGTCGTTYWTCA | AATGCAGTGACGTCGTTCTTCA |
|  | AATGCCGTCACGTCGTTTATCA |
| F:soilAbundant_amoA_AOB.C010  MTTCTACTGGTGGTCGCACTAC | ATTCTACTGGTGGTCGCACTAC |
|  | CTTCTACTGGTGGTCGCACTAC |
| R:soilAbundant_amoA_AOB.C010  KKAGYGASCCTTGTTCGATSWK | TGAGTGAGCCTTGTTCGATGTTG |
|  | GTAGTGAGCCTTGTTCGATCAGTC |
|  | GTAGCGACCCTTGTTCGATCAG |
| F:soilAbundant_amoA_AOB.C011  ACTACACCGGCTTCYTSTATRTA | ACTACACCGGCTTCCTGTATATAC |
|  | ACTACACCGGCTTCCTGTATGTA |
|  | ACTACACCGGCTTCCTCTATGTA |
|  | ACTACACCGGCTTCTTGTATGTAC |
| R:soilAbundant_amoA_AOB.C011  GTMRWYYTTSMYRRWRWMYCRS | GTAAACTTTGCCGAAATACCACCATAC |
|  | GTCGTTCTTCATGGTGACTCGG |
|  | GTCGTTCTTCATGGTAACCCGG |
|  | GTCGTTCTTCATAGTAACCCGGC |
| F:soilAbundant_amoA_AOB.C012  ATTTGGACTSYTGTTCTACCCK | ATTTGGACTGCTGTTCTACCCTG |
|  | ATTTGGACTCTTGTTCTACCCGG |
|  | ATTTGGACTCCTGTTCTACCCG |
|  | ATTTGGACTGTTGTTCTACCCTGG |
| R:soilAbundant_amoA_AOB.C012  AAAGTACCACCAKACGCAGAAC | AAAGTACCACCAGACGCAGAAC |
|  | AAAGTACCACCATACGCAGAACA |
| F:soilAbundant_amoA_AOB.C013  TGTGCWGCSWTSATGTAYTACY | TGTGCTGCCATCATGTACTACC |
|  | TGTGCAGCGTTGATGTATTACTTATG |
|  | TGTGCTGCGATCATGTACTACC |
| R:soilAbundant_amoA_AOB.C013  CCWGGRTAGAASAACAGDCCAAA | CCTGGGTAGAAGAACAGTCCAAAG |
|  | CCAGGATAGAACAACAGACCAAAGG |
|  | CCAGGATAGAACAACAGGCCAAA |
| F:soilAbundant_amoA_AOB.C014  GTVTTKCCCTCCACCATGATMC | GTATTGCCCTCCACCATGATACC |
|  | GTATTTCCCTCCACCATGATACCTG |
|  | GTCTTTCCCTCCACCATGATCC |
|  | GTGTTTCCCTCCACCATGATACC |
| R:soilAbundant_amoA_AOB.C014  CGYARYGAVCCTTGTTCGATCA | CGCAATGAACCTTGTTCGATCAG |
|  | CGTAGTGAGCCTTGTTCGATCA |
|  | CGTAACGACCCTTGTTCGATCA |
| F:soilAbundant_amoA_AOB.C015  GGGAYTTCTGSATKGACTGGAA | GGGATTTCTGCATTGACTGGAAA |
|  | GGGACTTCTGGATGGACTGGAA |
|  | GGGATTTCTGGATGGACTGGAA |
| R:soilAbundant_amoA_AOB.C015 | TCATGGTGGAGGGAAATACGAAG |
| F:soilAbundant_amoA_AOB.C016  TCAACTACMGWCTKCCCTTTGG | TCAACTACAGACTGCCCTTTGG |
|  | TCAACTACCGTCTTCCCTTTGG |
| R:soilAbundant_amoA_AOB.C016  GRTAGAACAGRAGTCCAAACGC | GGTAGAACAGGAGTCCAAACGC |
|  | GATAGAACAGAAGTCCAAACGCG |
|  | GGTAGAACAGAAGTCCAAACGC |
| F:soilAbundant_amoA_AOB.C017  HCTVYTGTTCTAYCCKGGYAAC | TCTATTGTTCTACCCGGGCAAC |
|  | CCTGCTGTTCTATCCTGGCAAC |
|  | ACTCCTGTTCTACCCGGGTAAC |
| R:soilAbundant_amoA_AOB.C017 | GTACCACCATACGCAGAACATGA |
| F:soilAbundant_amoA_AOB.C018  ACTGYKCCRCCATCATGTACTA | ACTGCTCCGCCATCATGTACTA |
|  | ACTGTGCCACCATCATGTACTAC |
| R:soilAbundant_amoA_AOB.C018  CAGWGCSGTGATCATCCAGTTR | CAGTGCGGTGATCATCCAGTTA |
|  | CAGAGCCGTGATCATCCAGTTG |
| F:soilAbundant_amoA_AOB.C019 | GATTCCTGGAGCACTGATCATGG |
| R:soilAbundant_amoA_AOB.C019 | GTAGCGACCCTTGTTCGATCAG |
| F:soilAbundant_amoA_AOB.C020  GYYTGTTGTTCTACCCTGGCAA | GTCTGTTGTTCTACCCTGGCAA |
|  | GCTTGTTGTTCTACCCTGGCAA |
| R:soilAbundant_amoA_AOB.C020  SACATAGTAGAAGGCGGTGCAG | CACATAGTAGAAGGCGGTGCAG |
|  | GACATAGTAGAAGGCGGTGCAG |
| F:soilAbundant_amoA_AOB.C021 | GTCAATGGTGGCCTGTAGTGAC |
| R:soilAbundant_amoA_AOB.C021  GGRTAGAACAGSAGGCCRAATG | GGATAGAACAGCAGGCCGAATG |
|  | GGGTAGAACAGGAGGCCAAATG |
| F:soilAbundant_amoA_AOB.C022 | GACTACACCGGCTTCCTGTATG |
| R:soilAbundant_amoA_AOB.C022 | GTAGAGTTTGCCGAGGTACCAC |
| F:soilAbundant_amoA_AOB.C023 | CTTCTGGCTGGACTGGAAAGAC |
| R:soilAbundant_amoA_AOB.C023 | GTATCATGGTGGAGGGCAAGAC |
| F:soilAbundant_amoA_AOB.C024 | GACTACACCGGCTTCCTGTATG |
| R:soilAbundant_amoA_AOB.C024 | GGTCACGTCGTTCTTCATGGATA |
| F:soilAbundant_amoA_AOB.C025 | CTTCTACTGGTGGTCGCACTAC |
| R:soilAbundant_amoA_AOB.C025 | AATGAGCCTTGTTCGATCAGCC |
| F:soilAbundant_amoA_AOB.C026 | ATGATACCTGGTGCACTGATGTTAG |
| R:soilAbundant_amoA_AOB.C026 | GTACCACCATACGCAGAACATCA |
| F:soilAbundant_amoA_AOB.C027 | GAAGCAGTAAAGATGTCCAGGTATATAGA |
| R:soilAbundant_amoA_AOB.C027 | GGCTCCAAAGGGCAGTCTATAG |
| F:soilAbundant_amoA_AOB.C028 | CTTACGCGCAACTGGATGATCA |
| R:soilAbundant_amoA_AOB.C028 | TTTGCCAAAGTACCACCAGACG |
