# Supplementary material for: MetaFunPrimer: an Environment-Specific, High-Throughput Primer Design Tool for Improved Quantification of Target Genes
Source: mSystems. 2021 Sep 21;6(5):e00201-21. doi: 10.1128/mSystems.00201-21 (PMC8547451; doi:10.1128/mSystems.00201-21)
Supplement: TABLE S4 [file msystems.00201-21-st004.docx]

**TABLE S4**Final set of primers for targeted *amo*A*-*AOB genes in soil metagenomes. Forward and reverse primer pairs have similar named identifiers, starting and ending in “F” for forward and “R” for reverse primer sequences.

| Forward primer | Forward sequence | Reverse Primer | Reverse sequence |
| --- | --- | --- | --- |
| F:amoA_AOB_p01F | CTTCTACTGGTGGTCGCACTAC | R:amoA_AOB_p01R | GTTCGATGTTGCGTACGTACTC |
| F:amoA_AOB_p02F | CTTCTACTGGTGGTCGCACTAC | R:amoA_AOB_p02R | TGAGTGAGCCTTGTTCGATGTTG |
| F:amoA_AOB_p03F | GACTACACCGGCTTCCTGTATG | R:amoA_AOB_p03R | AATGCAGTGACGTCGTTCTTCA |
| F:amoA_AOB_p04F | ACTCCTGTTCTACCCGGGTAAC | R:amoA_AOB_p04R | GTACCACCATACGCAGAACATGA |
| F:amoA_AOB_p05F | CTTCTACTGGTGGTCGCACTAC | R:amoA_AOB_p05R | GTTCGATCAGCCGTACGTACTC |
| F:amoA_AOB_p06F | GAAGCAGTAAAGATGTCCAGGTATATAGA | R:amoA_AOB_p06R | GGCTCCAAAGGGCAGTCTATAG |
| F:amoA_AOB_p07F | ACTACACCGGCTTCTTGTATGTAC | R:amoA_AOB_p07R | GTAAACTTTGCCGAAATACCACCATAC |
| F:amoA_AOB_p08F | GGCTTTCTGTATGTCCGTACCG | R:amoA_AOB_p08R | TCACGTCGTTCTTCATGCTGAC |
| F:amoA_AOB_p09F | TTTCTACTGGTGGTCGCACTAC | R:amoA_AOB_p09R | GTTCGATCAGACGCACGTACTC |
| F:amoA_AOB_p10F | GGCTTCCTGTATGTTCGTACCG | R:amoA_AOB_p10R | TCACGTCGTTCTTCATGCTGAC |
| F:amoA_AOB_p11F | CTTCTACTGGTGGTCGCACTAC | R:amoA_AOB_p11R | GTTCGATCAGACGCACGTACTC |
| F:amoA_AOB_p12F | GTCTGTTGTTCTACCCTGGCAA | R:amoA_AOB_p12R | GACATAGTAGAAGGCGGTGCAG |
| F:amoA_AOB_p13F | TGTGCTGCCATCATGTACTACC | R:amoA_AOB_p13R | CCAGGATAGAACAACAGGCCAAA |
| F:amoA_AOB_p14F | GGCTTCCTGTATGTACGTACCG | R:amoA_AOB_p14R | TCACGTCGTTCTTCATGCTGAC |
| F:amoA_AOB_p15F | TTTGGACTGTTGTTCTACCCTGG | R:amoA_AOB_p15R | TAGTAGAAGGCGGTGCAGTAGA |
| F:amoA_AOB_p16F | CTTCTACTGGTGGTCGCACTAC | R:amoA_AOB_p16R | GTTCGATCTGACGCACGTACTC |
| F:amoA_AOB_p17F | GGGACTTCTGGATTGACTGGAAAG | R:amoA_AOB_p17R | GGTATCATGGTGGAGGGAAATACG |
| F:amoA_AOB_p18F | GGGATTTCTGGCTTGACTGGAA | R:amoA_AOB_p18R | GGTATCATGGTGGAGGGAAATACG |
| F:amoA_AOB_p19F | CCCATCAACTTCGTATTTCCCTCC | R:amoA_AOB_p19R | TTCGATCAGCCGTACATACTCAG |
| F:amoA_AOB_p20F | ACTGCTCCGCCATCATGTACTA | R:amoA_AOB_p20R | CAGAGCCGTGATCATCCAGTTG |
| F:amoA_AOB_p21F | GACTACACCGGCTTCCTGTATG | R:amoA_AOB_p21R | AATGCCGTCACGTCGTTTATCA |
| F:amoA_AOB_p22F | TTTGGCCTGTTGTTCTACCCTG | R:amoA_AOB_p22R | TAGTAGAAGGCGGTGCAGTAGA |
| F:amoA_AOB_p23F | GACTACACCGGCTTCCTGTATG | R:amoA_AOB_p23R | GTGACGTCGTTCTTCATGCTGA |
| F:amoA_AOB_p24F | TTCGGTTTGTTGTTCTACCCTGG | R:amoA_AOB_p24R | TAGTAGAAGGCGGTGCAGTAGA |
| F:amoA_AOB_p25F | CTTACGCGCAACTGGATGATCA | R:amoA_AOB_p25R | TTTGCCAAAGTACCACCAGACG |
| F:amoA_AOB_p26F | GTATTTCCCTCCACCATGATACCTG | R:amoA_AOB_p26R | CGTAACGACCCTTGTTCGATCA |
| F:amoA_AOB_p27F | GTATTTCCCTCCACCATGATACCTG | R:amoA_AOB_p27R | CGCAATGAACCTTGTTCGATCAG |
| F:amoA_AOB_p28F | ATTTGGACTGTTGTTCTACCCTGG | R:amoA_AOB_p28R | AAAGTACCACCATACGCAGAACA |
| F:amoA_AOB_p29F | TTTGGTCTTCTGTTCTACCCGG | R:amoA_AOB_p29R | TAGTAGAAGGCGGTGCAGTAGA |
| F:amoA_AOB_p30F | TCAACTACCGTCTTCCCTTTGG | R:amoA_AOB_p30R | GATAGAACAGAAGTCCAAACGCG |
| F:amoA_AOB_p31F | TGTGCTGCGATCATGTACTACC | R:amoA_AOB_p31R | CCAGGATAGAACAACAGACCAAAGG |
| F:amoA_AOB_p32F | TGTGCTGCGATCATGTACTACC | R:amoA_AOB_p32R | CCAGGATAGAACAACAGGCCAAA |
| F:amoA_AOB_p33F | CCTGCTGTTCTATCCTGGCAAC | R:amoA_AOB_p33R | GTACCACCATACGCAGAACATGA |
| F:amoA_AOB_p34F | TCTATTGTTCTACCCGGGCAAC | R:amoA_AOB_p34R | GTACCACCATACGCAGAACATGA |
| F:amoA_AOB_p35F | GCTGCCATCATGTACTACCTGTG | R:amoA_AOB_p35R | CAGGGCTGTAACCATCCAGTTG |
| F:amoA_AOB_p36F | GATTACACCGGCTTCCTGTATGTAC | R:amoA_AOB_p36R | AATGCAGTGACGTCGTTCTTCA |
| F:amoA_AOB_p37F | ACTGTGCCACCATCATGTACTAC | R:amoA_AOB_p37R | CAGTGCGGTGATCATCCAGTTA |
| F:amoA_AOB_p38F | TCAACTACAGACTGCCCTTTGG | R:amoA_AOB_p38R | GGTAGAACAGGAGTCCAAACGC |
| F:amoA_AOB_p39F | GCTTGTTGTTCTACCCTGGCAA | R:amoA_AOB_p39R | CACATAGTAGAAGGCGGTGCAG |
| F:amoA_AOB_p40F | ATTTGGACTGCTGTTCTACCCTG | R:amoA_AOB_p40R | AAAGTACCACCATACGCAGAACA |
| F:amoA_AOB_p41F | GATTCCTGGAGCACTGATCATGG | R:amoA_AOB_p41R | GTAGCGACCCTTGTTCGATCAG |
| F:amoA_AOB_p42F | CCCATCAACTTCGTATTTCCCTCC | R:amoA_AOB_p42R | TTCGATCAGCCTCACGTACTCA |
| F:amoA_AOB_p43F | GTGTTTCCCTCCACCATGATACC | R:amoA_AOB_p43R | CGTAACGACCCTTGTTCGATCA |
| F:amoA_AOB_p44F | GACTACACCGGCTTCCTGTATG | R:amoA_AOB_p44R | GTCACGTCGTTCTTCATGGAGA |
| F:amoA_AOB_p45F | GTCAATGGTGGCCTGTAGTGAC | R:amoA_AOB_p45R | GGATAGAACAGCAGGCCGAATG |
| F:amoA_AOB_p46F | GTCAATGGTGGCCTGTAGTGAC | R:amoA_AOB_p46R | GGGTAGAACAGGAGGCCAAATG |
| F:amoA_AOB_p47F | GACTACACCGGCTTTCTGTATGTAC | R:amoA_AOB_p47R | GTCACGTCGTTCTTCATGGAGA |
| F:amoA_AOB_p48F | TCAACTACCGTCTTCCCTTTGG | R:amoA_AOB_p48R | GGTAGAACAGAAGTCCAAACGC |
| F:amoA_AOB_p49F | GACTACACCGGCTTTCTGTATGTAC | R:amoA_AOB_p49R | GTAACGTCGTTCTGCATGGAGA |
| F:amoA_AOB_p50F | AGCTTTCTGTATGTACGCACGG | R:amoA_AOB_p50R | TAACGTCGTTCTGCATGGAGATG |
| F:amoA_AOB_p51F | GTATTGCCCTCCACCATGATACC | R:amoA_AOB_p51R | CGTAGTGAGCCTTGTTCGATCA |
| F:amoA_AOB_p52F | GCCACCATCATGTACTACCTGTG | R:amoA_AOB_p52R | CAGTGCTGTGATCATCCAGTTG |
| F:amoA_AOB_p53F | CTTCTACTGGTGGTCGCACTAC | R:amoA_AOB_p53R | GTTCGATCAGCCGTACGTATTCC |
| F:amoA_AOB_p54F | TTTGGACTGTTGTTCTACCCGG | R:amoA_AOB_p54R | TAGTAGAACGCGGTGCAGTAGA |
| F:amoA_AOB_p55F | GACTACACCGGCTTCCTGTATG | R:amoA_AOB_p55R | GTGACGTCGTTCTTCATGGTAAC |
| F:amoA_AOB_p56F | CTTCTGGCTGGACTGGAAAGAC | R:amoA_AOB_p56R | GTATCATGGTGGAGGGCAAGAC |
| F:amoA_AOB_p57F | CTTCTACTGGTGGTCGCACTAC | R:amoA_AOB_p57R | GTAGTGAGCCTTGTTCGATCAGTC |
| F:amoA_AOB_p58F | GACTACACCGGCTTCCTGTATG | R:amoA_AOB_p58R | GTAACGTCGTTCTTCATGGTGAC |
| F:amoA_AOB_p59F | ACTACACCGGCTTCCTGTATGTA | R:amoA_AOB_p59R | GTCGTTCTTCATGGTGACTCGG |
| F:amoA_AOB_p60F | ACTTCTGGCTGGACTGGAAAGA | R:amoA_AOB_p60R | AATATCATGGTGGAGGGCAAGAC |
| F:amoA_AOB_p61F | ACTACACCGGCTTCCTCTATGTA | R:amoA_AOB_p61R | GTCGTTCTTCATGGTGACTCGG |
| F:amoA_AOB_p62F | ATTTGGACTCCTGTTCTACCCG | R:amoA_AOB_p62R | AAAGTACCACCAGACGCAGAAC |
| F:amoA_AOB_p63F | GGGATTTCTGGATGGACTGGAA | R:amoA_AOB_p63R | GGTATCATGGTGGAGGGCAATA |
| F:amoA_AOB_p64F | GGGATTTCTGGATGGACTGGAA | R:amoA_AOB_p64R | GGTATCATGGTGGAGGGCAGTA |
| F:amoA_AOB_p65F | ATTTGGACTCTTGTTCTACCCGG | R:amoA_AOB_p65R | AAAGTACCACCAGACGCAGAAC |
| F:amoA_AOB_p66F | CTTCTACTGGTGGTCGCACTAC | R:amoA_AOB_p66R | AATGAGCCTTGTTCGATCAGCC |
| F:amoA_AOB_p67F | GGGACTTCTGGATGGACTGGAA | R:amoA_AOB_p67R | TCATGGTGGAGGGAAATACGAAG |
| F:amoA_AOB_p68F | GACTACACCGGCTTCCTGTATG | R:amoA_AOB_p68R | GTAGAGTTTGCCGAGGTACCAC |
| F:amoA_AOB_p69F | GACTACACCGGCTTCCTGTATG | R:amoA_AOB_p69R | GGTCACGTCGTTCTTCATGGATA |
| F:amoA_AOB_p70F | CCCATCAACTTCGTCTTTCCCTC | R:amoA_AOB_p70R | TTCGATCAGGCGCACATATTCAG |
| F:amoA_AOB_p71F | ACTACACCGGCTTCCTGTATATAC | R:amoA_AOB_p71R | GTCGTTCTTCATGGTAACCCGG |
| F:amoA_AOB_p72F | GTCTTTCCCTCCACCATGATCC | R:amoA_AOB_p72R | CGTAACGACCCTTGTTCGATCA |
| F:amoA_AOB_p73F | GGGATTTCTGCATTGACTGGAAA | R:amoA_AOB_p73R | TCATGGTGGAGGGAAATACGAAG |
| F:amoA_AOB_p74F | GGGATTTCTGGATGGACTGGAA | R:amoA_AOB_p74R | GGTATCATGGTGGAGGGAAATACG |
| F:amoA_AOB_p75F | TGTGCAGCGTTGATGTATTACTTATG | R:amoA_AOB_p75R | CCTGGGTAGAAGAACAGTCCAAAG |
| F:amoA_AOB_p76F | GCGGCGTTGATGTATTATTTGTGG | R:amoA_AOB_p76R | TAATGCCGTTACCAGCCAGTTAC |
| F:amoA_AOB_p77F | CCGATCAACTTTGTACTGCCATC | R:amoA_AOB_p77R | CTCAATCAGGCGAACATATTCCG |
| F:amoA_AOB_p78F | ATGATACCTGGTGCACTGATGTTAG | R:amoA_AOB_p78R | GTACCACCATACGCAGAACATCA |
