# Supplementary material for: MetaFunPrimer: an Environment-Specific, High-Throughput Primer Design Tool for Improved Quantification of Target Genes
Source: mSystems. 2021 Sep 21;6(5):e00201-21. doi: 10.1128/mSystems.00201-21 (PMC8547451; doi:10.1128/mSystems.00201-21)
Supplement: TABLE S5 [file msystems.00201-21-st005.docx]

**TABLE S5**Previously published *amo*A-AOB primer pairs that were used to compare with the newly designed primer pairs in this study. These primer pairs have been widely used in literature.

| **Previously published primer** | **Sequence (5’ to 3’)** | **Reference** |
| --- | --- | --- |
| amoA-1FF | CAATGGTGGCCGGTTGT | Hoshino et al., 2001 (1) |
| amoA-2R | CCCCTCKGSAAAGCCTTCTTC | Rotthauwe et al., 1997 (2) |
| amoA-1F | GGGGTTTCTACTGGTGGT | Rotthauwe et al., 1997 (2) |
| amoA-2R | CCCCTCKGSAAAGCCTTCTTC | Rotthauwe et al., 1997 (2) |
| amoA-1F* | GGGGHTTYTACTGGTGGT | Stephen et al., 1999 (3) |
| amoA-2R | CCCCTCKGSAAAGCCTTCTTC | Rotthauwe et al., 1997 (2) |
| amoA-1Fmod | CTGGGGTTTCTACTGGTGGTC | Meinhardt et al., 2015 (4) |
| GenAOBR | GCAGTGATCATCCAGTTGCG | Meinhardt et al., 2015 (4) |

**REFERENCES**

1. Hoshino T, Noda N, Tsuneda S, Hirata A, Inamori Y. 2001. Direct Detection by in Situ PCR of the amoA Gene in Biofilm Resulting from a Nitrogen Removal Process. Appl Environ Microbiol 67:5261–5266.

2. Rotthauwe JH, Witzel KP, Liesack W. 1997. The ammonia monooxygenase structural gene amoa as a functional marker: Molecular fine-scale analysis of natural ammonia-oxidizing populations. Appl Environ Microbiol 63:4704–4712.

3. Stephen JR, Chang YJ, Macnaughton SJ, Kowalchuk GA, Leung KT, Flemming CA, White DC. 1999. Effect of toxic metals on indigenous soil β-subgroup proteobacterium ammonia oxidizer community structure and protection against toxicity by inoculated metal-resistant bacteria. Appl Environ Microbiol 65:95–101.

4. Meinhardt KA, Bertagnolli A, Pannu MW, Strand SE, Brown SL, Stahl DA. 2015. Evaluation of revised polymerase chain reaction primers for more inclusive quantification of ammonia-oxidizing archaea and bacteria. Environ Microbiol Rep 7:354–363.
