# Supplementary material for: MetaFunPrimer: an Environment-Specific, High-Throughput Primer Design Tool for Improved Quantification of Target Genes
Source: mSystems. 2021 Sep 21;6(5):e00201-21. doi: 10.1128/mSystems.00201-21 (PMC8547451; doi:10.1128/mSystems.00201-21)
Supplement: TABLE S6 [file msystems.00201-21-st006.docx]

**TABLE S6** The quantities of *amo*A-AOB genes enumerated using each primer set (unit: gene copies per gram soil). Amplifications below the detection limit (5.46 × 10^6^ gene copies per gram soil) were neglected.

| Sample | amoA_AOB_p12 | amoA_AOB_p31 | amoA_AOB_p35 | amoA_AOB_p42 | amoA_AOB_p45 | amoA_AOB_p70 |
| --- | --- | --- | --- | --- | --- | --- |
| 37b | 5.03E+08 | 5.05E+07 | 1.07E+08 | 0.00E+00 | 0.00E+00 | 0.00E+00 |
| 37f | 3.79E+08 | 4.39E+07 | 9.59E+07 | 0.00E+00 | 0.00E+00 | 0.00E+00 |
| 38b | 4.56E+08 | 4.52E+07 | 1.24E+08 | 0.00E+00 | 0.00E+00 | 0.00E+00 |
| 38f | 4.30E+08 | 3.06E+07 | 5.47E+07 | 0.00E+00 | 0.00E+00 | 0.00E+00 |
| 39b | 7.15E+08 | 4.77E+07 | 1.67E+08 | 0.00E+00 | 0.00E+00 | 0.00E+00 |
| 39f | 3.01E+08 | 0.00E+00 | 5.94E+07 | 0.00E+00 | 0.00E+00 | 0.00E+00 |
| 43b | 1.29E+08 | 0.00E+00 | 0.00E+00 | 1.13E+07 | 1.08E+08 | 7.02E+06 |
| 43f | 1.01E+08 | 0.00E+00 | 0.00E+00 | 6.91E+06 | 4.03E+07 | 6.10E+06 |
| 44b | 5.60E+07 | 0.00E+00 | 0.00E+00 | 1.12E+07 | 4.98E+07 | 1.14E+07 |
| 44f | 8.68E+07 | 0.00E+00 | 0.00E+00 | 0.00E+00 | 6.76E+07 | 0.00E+00 |
| 45b | 1.23E+08 | 0.00E+00 | 0.00E+00 | 0.00E+00 | 9.39E+07 | 8.39E+06 |
| 45f | 9.33E+07 | 0.00E+00 | 0.00E+00 | 0.00E+00 | 3.64E+07 | 7.28E+06 |
| 49b | 0.00E+00 | 0.00E+00 | 0.00E+00 | 0.00E+00 | 0.00E+00 | 0.00E+00 |
| 49f | 2.59E+08 | 0.00E+00 | 2.48E+07 | 0.00E+00 | 0.00E+00 | 0.00E+00 |
| 50b | 1.25E+08 | 0.00E+00 | 1.70E+07 | 0.00E+00 | 0.00E+00 | 0.00E+00 |
| 50f | 7.80E+07 | 0.00E+00 | 2.04E+07 | 0.00E+00 | 2.21E+07 | 0.00E+00 |
| 51b | 1.52E+08 | 0.00E+00 | 2.24E+07 | 0.00E+00 | 1.11E+07 | 0.00E+00 |
| 51f | 1.10E+08 | 0.00E+00 | 4.48E+07 | 0.00E+00 | 0.00E+00 | 0.00E+00 |
| 52b | 1.34E+08 | 0.00E+00 | 0.00E+00 | 0.00E+00 | 2.82E+07 | 0.00E+00 |
| 52f | 7.76E+07 | 0.00E+00 | 0.00E+00 | 0.00E+00 | 4.58E+07 | 0.00E+00 |
| 53b | 1.28E+08 | 0.00E+00 | 0.00E+00 | 0.00E+00 | 2.15E+07 | 0.00E+00 |
| 53f | 9.99E+07 | 0.00E+00 | 0.00E+00 | 0.00E+00 | 5.03E+07 | 0.00E+00 |
| 54b | 1.50E+08 | 0.00E+00 | 0.00E+00 | 0.00E+00 | 5.01E+07 | 0.00E+00 |
| 54f | 4.28E+07 | 0.00E+00 | 0.00E+00 | 0.00E+00 | 1.00E+07 | 0.00E+00 |
| 55b | 4.18E+08 | 4.49E+07 | 8.05E+07 | 0.00E+00 | 0.00E+00 | 0.00E+00 |
| 55f | 5.14E+08 | 3.40E+07 | 9.58E+07 | 0.00E+00 | 0.00E+00 | 0.00E+00 |
| 56b | 5.14E+08 | 2.41E+07 | 1.44E+08 | 0.00E+00 | 0.00E+00 | 0.00E+00 |
| 56f | 1.43E+08 | 2.03E+07 | 2.47E+07 | 0.00E+00 | 0.00E+00 | 0.00E+00 |
| 57b | 5.90E+08 | 6.46E+07 | 1.49E+08 | 0.00E+00 | 0.00E+00 | 0.00E+00 |
| 57f | 3.92E+08 | 2.41E+07 | 4.86E+07 | 0.00E+00 | 0.00E+00 | 0.00E+00 |
| 61b | 1.68E+08 | 1.43E+07 | 0.00E+00 | 0.00E+00 | 0.00E+00 | 0.00E+00 |
| 61f | 2.24E+08 | 0.00E+00 | 1.83E+07 | 0.00E+00 | 0.00E+00 | 0.00E+00 |
| 62b | 2.04E+08 | 0.00E+00 | 2.02E+07 | 0.00E+00 | 0.00E+00 | 0.00E+00 |
| 62f | 0.00E+00 | 0.00E+00 | 0.00E+00 | 0.00E+00 | 0.00E+00 | 0.00E+00 |
| 63b | 1.28E+08 | 0.00E+00 | 2.86E+07 | 0.00E+00 | 0.00E+00 | 0.00E+00 |
| 63f | 1.15E+08 | 0.00E+00 | 2.26E+07 | 0.00E+00 | 0.00E+00 | 0.00E+00 |
| 64b | 2.75E+08 | 0.00E+00 | 0.00E+00 | 1.95E+07 | 5.15E+07 | 1.45E+07 |
| 64f | 2.91E+08 | 0.00E+00 | 1.87E+07 | 1.93E+07 | 5.43E+07 | 1.32E+07 |
| 65b | 2.38E+08 | 0.00E+00 | 2.12E+07 | 1.19E+07 | 1.56E+08 | 6.43E+06 |
| 65f | 4.54E+07 | 0.00E+00 | 0.00E+00 | 9.06E+06 | 5.88E+07 | 1.21E+07 |
| 66b | 2.20E+08 | 0.00E+00 | 1.78E+07 | 1.86E+07 | 5.07E+07 | 1.35E+07 |
| 66f | 1.57E+08 | 0.00E+00 | 0.00E+00 | 5.69E+06 | 1.02E+08 | 9.10E+06 |
| 67b | 1.40E+08 | 0.00E+00 | 2.53E+07 | 0.00E+00 | 0.00E+00 | 0.00E+00 |
| 67f | 1.66E+08 | 0.00E+00 | 1.42E+07 | 0.00E+00 | 0.00E+00 | 0.00E+00 |
| 68b | 1.39E+08 | 0.00E+00 | 0.00E+00 | 0.00E+00 | 0.00E+00 | 0.00E+00 |
| 68f | 1.24E+08 | 0.00E+00 | 2.19E+07 | 0.00E+00 | 0.00E+00 | 0.00E+00 |
| 69b | 8.39E+07 | 0.00E+00 | 0.00E+00 | 0.00E+00 | 0.00E+00 | 0.00E+00 |
| 69f | 9.36E+07 | 0.00E+00 | 0.00E+00 | 0.00E+00 | 0.00E+00 | 0.00E+00 |
| 13b | 6.42E+07 | 0.00E+00 | 0.00E+00 | 0.00E+00 | 5.98E+07 | 1.15E+07 |
| 13f | 1.08E+08 | 0.00E+00 | 0.00E+00 | 0.00E+00 | 5.75E+07 | 0.00E+00 |
| 14b | 1.17E+08 | 0.00E+00 | 0.00E+00 | 6.45E+06 | 1.44E+08 | 5.98E+06 |
| 14f | 1.24E+08 | 0.00E+00 | 0.00E+00 | 7.69E+06 | 2.69E+07 | 0.00E+00 |
| 15b | 1.29E+08 | 0.00E+00 | 0.00E+00 | 7.95E+06 | 6.02E+07 | 1.12E+07 |
| 15f | 5.28E+07 | 0.00E+00 | 0.00E+00 | 0.00E+00 | 5.29E+07 | 0.00E+00 |
| 16b | 1.90E+08 | 0.00E+00 | 0.00E+00 | 1.33E+07 | 6.39E+07 | 1.41E+07 |
| 16f | 1.20E+08 | 0.00E+00 | 5.16E+07 | 0.00E+00 | 1.09E+08 | 7.05E+06 |
| 17b | 1.62E+08 | 0.00E+00 | 2.20E+07 | 6.53E+06 | 1.75E+08 | 6.63E+06 |
| 17f | 1.34E+08 | 0.00E+00 | 0.00E+00 | 0.00E+00 | 5.05E+07 | 9.59E+06 |
| 18b | 1.85E+08 | 0.00E+00 | 0.00E+00 | 8.25E+06 | 1.96E+08 | 8.43E+06 |
| 18f | 1.56E+08 | 0.00E+00 | 0.00E+00 | 1.22E+07 | 3.50E+07 | 1.09E+07 |
| 1b | 2.25E+08 | 0.00E+00 | 4.13E+07 | 0.00E+00 | 0.00E+00 | 0.00E+00 |
| 1f | 1.42E+08 | 0.00E+00 | 0.00E+00 | 0.00E+00 | 0.00E+00 | 0.00E+00 |
| 22b | 3.15E+08 | 4.33E+07 | 1.56E+07 | 0.00E+00 | 0.00E+00 | 0.00E+00 |
| 22f | 0.00E+00 | 0.00E+00 | 0.00E+00 | 0.00E+00 | 0.00E+00 | 0.00E+00 |
| 23b | 2.24E+08 | 1.73E+07 | 3.90E+07 | 0.00E+00 | 0.00E+00 | 0.00E+00 |
| 23f | 2.23E+08 | 1.99E+07 | 3.57E+07 | 0.00E+00 | 0.00E+00 | 0.00E+00 |
| 24b | 3.23E+08 | 3.62E+07 | 1.38E+07 | 0.00E+00 | 0.00E+00 | 0.00E+00 |
| 24f | 2.98E+08 | 4.00E+07 | 3.03E+07 | 0.00E+00 | 0.00E+00 | 0.00E+00 |
| 25b | 3.56E+08 | 7.96E+07 | 6.82E+07 | 0.00E+00 | 0.00E+00 | 0.00E+00 |
| 25f | 3.10E+08 | 8.80E+07 | 4.04E+07 | 0.00E+00 | 0.00E+00 | 0.00E+00 |
| 26b | 3.08E+08 | 7.25E+07 | 6.87E+07 | 0.00E+00 | 0.00E+00 | 0.00E+00 |
| 26f | 3.47E+08 | 3.39E+07 | 6.87E+07 | 0.00E+00 | 0.00E+00 | 0.00E+00 |
| 27b | 3.72E+08 | 2.63E+07 | 6.21E+07 | 0.00E+00 | 0.00E+00 | 0.00E+00 |
| 27f | 3.12E+08 | 1.25E+07 | 6.33E+07 | 0.00E+00 | 3.20E+07 | 0.00E+00 |
| 28b | 9.74E+07 | 0.00E+00 | 4.07E+07 | 0.00E+00 | 0.00E+00 | 0.00E+00 |
| 28f | 1.55E+08 | 0.00E+00 | 2.57E+07 | 0.00E+00 | 1.85E+07 | 0.00E+00 |
| 29b | 9.09E+07 | 0.00E+00 | 3.82E+07 | 0.00E+00 | 0.00E+00 | 0.00E+00 |
| 29f | 1.11E+08 | 0.00E+00 | 3.06E+07 | 0.00E+00 | 0.00E+00 | 0.00E+00 |
| 2b | 1.41E+08 | 0.00E+00 | 2.65E+07 | 0.00E+00 | 0.00E+00 | 0.00E+00 |
| 2f | 1.62E+08 | 0.00E+00 | 3.17E+07 | 0.00E+00 | 0.00E+00 | 0.00E+00 |
| 30b | 2.48E+08 | 0.00E+00 | 0.00E+00 | 0.00E+00 | 0.00E+00 | 0.00E+00 |
| 30f | 1.51E+08 | 0.00E+00 | 1.19E+07 | 0.00E+00 | 0.00E+00 | 0.00E+00 |
| 34b | 9.77E+07 | 0.00E+00 | 2.94E+07 | 0.00E+00 | 2.58E+07 | 0.00E+00 |
| 34f | 8.45E+07 | 0.00E+00 | 0.00E+00 | 0.00E+00 | 3.25E+07 | 0.00E+00 |
| 35b | 1.04E+08 | 0.00E+00 | 2.41E+07 | 0.00E+00 | 0.00E+00 | 0.00E+00 |
| 35f | 0.00E+00 | 0.00E+00 | 0.00E+00 | 0.00E+00 | 0.00E+00 | 0.00E+00 |
| 36b | 8.45E+07 | 0.00E+00 | 0.00E+00 | 0.00E+00 | 0.00E+00 | 0.00E+00 |
| 36f | 8.84E+07 | 0.00E+00 | 0.00E+00 | 0.00E+00 | 0.00E+00 | 0.00E+00 |
| 3b | 1.70E+08 | 0.00E+00 | 4.04E+07 | 0.00E+00 | 0.00E+00 | 0.00E+00 |
| 3f | 1.74E+08 | 0.00E+00 | 0.00E+00 | 0.00E+00 | 0.00E+00 | 0.00E+00 |
| 7b | 1.30E+08 | 0.00E+00 | 0.00E+00 | 0.00E+00 | 1.62E+07 | 0.00E+00 |
| 7f | 8.79E+07 | 0.00E+00 | 0.00E+00 | 0.00E+00 | 0.00E+00 | 0.00E+00 |
| 8b | 1.28E+08 | 0.00E+00 | 2.16E+07 | 0.00E+00 | 3.75E+07 | 0.00E+00 |
| 8f | 1.46E+08 | 0.00E+00 | 0.00E+00 | 0.00E+00 | 3.65E+07 | 0.00E+00 |
| 9b | 1.35E+08 | 0.00E+00 | 0.00E+00 | 0.00E+00 | 6.78E+07 | 0.00E+00 |
| 9f | 1.55E+08 | 0.00E+00 | 2.13E+07 | 0.00E+00 | 0.00E+00 | 0.00E+00 |
