# Supplementary material for: MetaFunPrimer: an Environment-Specific, High-Throughput Primer Design Tool for Improved Quantification of Target Genes
Source: mSystems. 2021 Sep 21;6(5):e00201-21. doi: 10.1128/mSystems.00201-21 (PMC8547451; doi:10.1128/mSystems.00201-21)
Supplement: TABLE S7 [file msystems.00201-21-st007.docx]

**TABLE S7**Parameters used for primer design for *amo*A-AOB in this study.

| **Parameters** | **Default settings** | **Recommend range** |
| --- | --- | --- |
| Minimum amplicon product length | 220 bp |  |
| Maximum amplicon product length | 330 bp |  |
| Minimum oligo size | 22 bp | no shorter than 15 bp |
| Maximum oligo size | 30 bp | no longer than 30 bp |
| Minimum oligo melting temperature | 59 Celsius |  |
| Maximum oligo melting temperature | 61 Celsius |  |
| Hairpin maximum temperature | 24 Celsius |  |
| Homodimer maximum temperature | 35 Celsius |  |
| Maximum degeneracy per primer pair | 6 | no higher than 10 |
| Maximum number of assays allowed | 30 |  |
| (one degenerate primer pair per assay) |  |  |
| G+C content filter minimum percent | 0.15 |  |
| G+C content filter maximum percent | 0.8 |  |
| Maximum oligo mismatch | 0 | 0-3 |
| isTreeWeightNeeded | f |  |
| isHenikoffWeightNeeded | f |  |
| os | Linux |  |
| NoTEndFilter | t |  |
| NoPoly3GCFilter | t |  |
| PolyRunFilter | 4 |  |
