# Supplementary material for: MetaFunPrimer: an Environment-Specific, High-Throughput Primer Design Tool for Improved Quantification of Target Genes
Source: mSystems. 2021 Sep 21;6(5):e00201-21. doi: 10.1128/mSystems.00201-21 (PMC8547451; doi:10.1128/mSystems.00201-21)
Supplement: FIG S2 [file msystems.00201-21-sf002.docx]

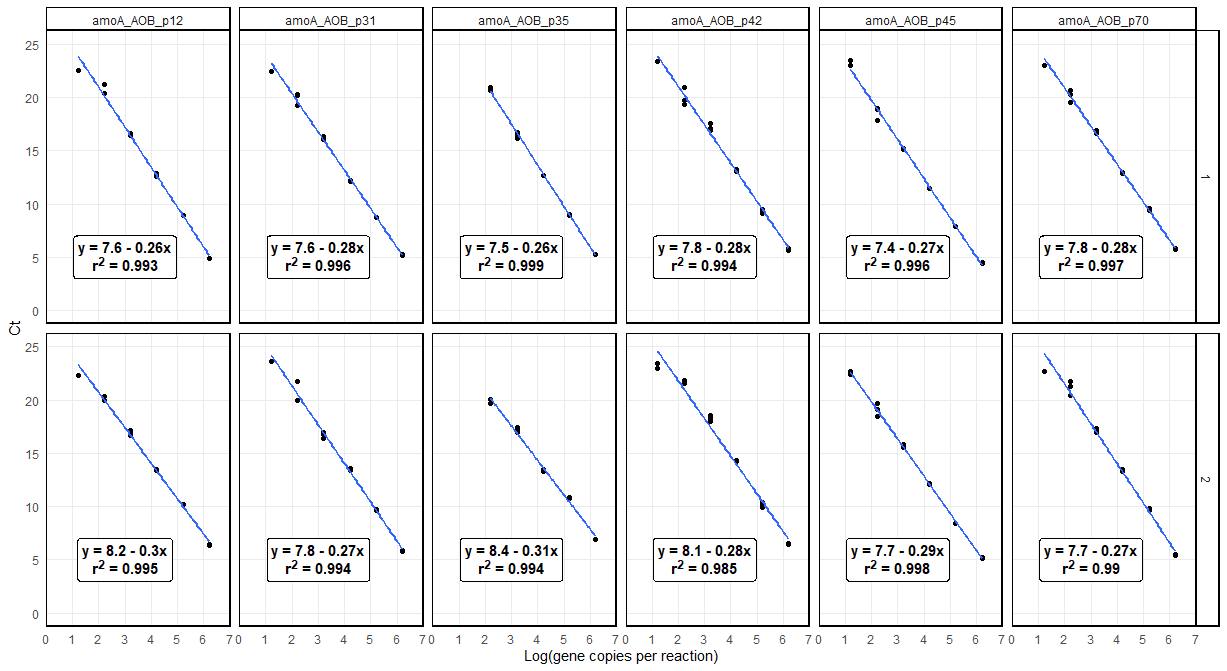


**Fig. S2** Standard curves (a plot of the log gene copies per reaction versus Ct value of standard DNA samples) for the 6 primer pairs identified for absolute quantification separated by first and second HT-qPCR runs. The blue line indicates the line of best calculated via the lm function in R. The equation for each of these lines and the associated r^2^ is in each plot.
