# Supplementary material for: MetaFunPrimer: an Environment-Specific, High-Throughput Primer Design Tool for Improved Quantification of Target Genes
Source: mSystems. 2021 Sep 21;6(5):e00201-21. doi: 10.1128/mSystems.00201-21 (PMC8547451; doi:10.1128/mSystems.00201-21)
Supplement: TEXT S1 [file msystems.00201-21-s0001.docx]

**TEXT S1**Biomark assay conditions for each Integrated Fluidic Circuit (IFC).

| IFC | Reaction vol. (nL) | Primer conc. (nM) | Assay mix for each primer set (uL) | | | Sample mix for each sample (uL) | | |
| --- | --- | --- | --- | --- | --- | --- | --- | --- |
|  |  |  | Loading | Buffer | Primer | EvaGreen | Binding | Sample |
| Flex Six | 8.9 | 500 | 2.5 | 2 | 0.5 | 2.5 | 0.25 | 2.25 |
| 96.96 | 6.7 | 500 | 1.5 | 1.2 | 0.3 | 1.5 | 0.15 | 1.35 |

A. Reagent and materials for Flex Six assays

1) 2X Assay Loading Reagent (Fluidigm PN 100-7611)

2) 1X DNA Suspension Buffer (TEKnova PN T0221)

3) 50 μM combined forward and reverse primers

4) 2X SsoFast EvaGreen Supermix with low ROX (BioRad PN 172-5211)

5) Flex Six Delta Gene Sample Reagent (Fluidigm PN 100-7673) (Red_cap)

6) 10-fold diluted soil DNA samples and standard DNA samples

B. Reagent and materials for 96.96 assays

1) 2X Assay Loading Reagent (Fluidigm PN 100-7611)

2) 1X DNA Suspension Buffer (TEKnova PN T0221)

3) 50 μM combined forward and reverse primers

4) 2X SsoFast EvaGreen Supermix with low ROX (BioRad PN 172-5211)

5) 20X DNA Binding Dye (Fluidigm PN 100-7609)

6) 10-fold diluted soil DNA samples
